# Dehydrogenation and dehydration of formic acid over orthorhombic molybdenum carbide

**DOI:** 10.1016/j.cattod.2021.04.011

**Published:** 2022-02-15

**Authors:** Kushagra Agrawal, Alberto Roldan, Nanda Kishore, Andrew J. Logsdail

**Affiliations:** a Department of Chemical Engineering, Indian Institute of Technology Guwahati, Guwahati, 781039, Assam, India; b Cardiff Catalysis Institute, School of Chemistry, Cardiff University, Park Place, Cardiff, CF10 3AT, Wales, UK

**Keywords:** Mo_2_C, Molybdenum carbide, Formic acid, Hydrodeoxygenation, Heterogeneous catalysis, Bio-oil

## Abstract

The dehydrogenation and dehydration of formic acid is investigated on the *β*-Mo_2_C (100) catalyst surface using time independent density functional theory. The energetics of the two mechanisms are calculated, and the thermochemistry and kinetics are discussed using the transition state theory. Subsequently, microkinetic modelling of the system is conducted, considering the batch reactor model. The potential energy landscape of the reaction shows a thermodynamically favourable cleavage of H—COOH to form CO; however, the kinetics show that the dehydrogenation mechanism is faster and CO_2_ is continuously formed. The effect of HCOOH adsorption on the surface is also analysed, in a temperature-programmed desorption, with the conversion proceeding at under 350 K and desorption of CO_2_ is observed with a selectivity of about 100 %, in line with the experimental reports.

## Highlights

• Formic acid (HCOOH) adsorption on *β*-Mo_2_C is exothermic and favours a configuration parallel to the surface.
• Once adsorbed, thermodynamics favour cleavage of the H—COOH bond to form CO.
• CO bonds strongly to the surface, potentially poisoning the catalyst.
• Therefore, kinetics favour dehydrogenation mechanism with CO_2_ continuously formed.

## Graphical abstract

**Figure:**
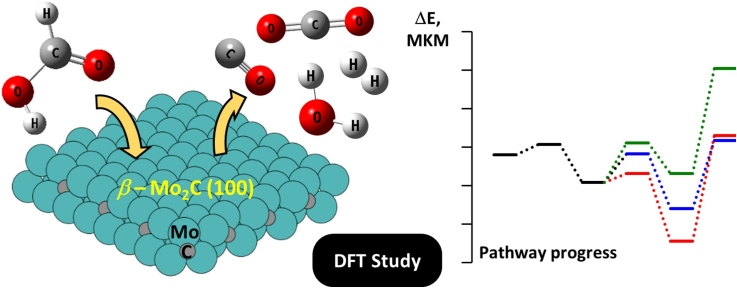

## 1 Introduction

In the continued efforts to identify renewable and portable energy sources that sustain modern society, biomass is a promising future candidate. Biomass can be converted to carbon-based bio-fuel, which has created considerable interest across academia and industry [1]; however, the economics of producing transport-grade bio-oil does not yet make commercial applications viable. Problems associated with chlorine induced corrosion in the biomass boiler [2], and the presence of tar at the gasifier outlet [3] are particularly major challenges that must be addressed prior to broader uptake, and widespread exploitation of bio-oils is prevented because the process of hydrodeoxygenation (HDO), which is a crucial step in upgrade the bio-oil energy density [4], is energy demanding and costly. Indeed, biomass typically has large quantities of oxy-compounds that necessitate HDO treatment to improve the bio-oil quality.

Catalysts accelerate chemical reactions and lower production costs [5], and thus many different materials are being investigation as catalysts for the HDO process. Transition metals like platinum, palladium and ruthenium are good HDO catalysts [4], but their high cost, due to their scarcity, hinders industrial implementation; however, the beneficial properties of the active catalysts can be used to design new catalysts composed of earth-abundant elements. For precious metals, the positioning of their *d*-band electronic states correlates significantly with the catalytic activity [6]. The importance of the *d*-band can be rationalised thus: as an adsorbate approaches the catalytic surface, the *s* and *d* orbitals of the metal catalyst interact with the frontier orbitals of the adsorbate, forming both bonding and anti-bonding states; the relative energy of the anti-bonding state, compared to the centre of the *d*-band, then determine the strength of the bond between the surface and the adsorbate. Therefore, the *d*-band centre correlates with reactivity [7]. Although the high Pt-like reactivity of metal carbides has been known for a long time [8], it is only recently that molybdenum carbide (Mo_2_C) has been identified to have similar *d*-band characteristics to that of noble metals [9]. This electronic character, together with its thermal stability, offers an exciting opportunity for application of this lower cost material in domains that have been previously limited to precious metals. The nature of the *d-*band centre in Mo_2_C, and specifically its contraction to favour catalytic applications, arises due to the increase in the interatomic bond length of the Mo lattice as it is carburized [10]. As a result of the *d-*band contraction, the *d*-electron states are increased at the Fermi level, akin to the density of states observed for noble metals [11].

Mo_2_C has been applied as a catalyst in the HDO process in several experimental investigations. Mai et al. [12] studied the conversion of levulinic acid to γ-valerolactone catalysed by carbon nanotube (CNT) supported molybdenum carbide, identifying that the CNT support is crucial to prevent catalyst deactivation; at operating conditions of 30 bar H_2_ and 200 °C, the conversion and selectivity with this system is >90 %, which is similar to the performance of noble metals. Han et al. [13] upgraded a range of different vegetable oils (soybean oil, rapeseed oil, maize oil, sunflower oil) to transport-grade fuel over a molybdenum carbide catalyst, reporting an ability to recycle the catalyst 16 times for the upgrading process with no leaching observed; however, the product distribution with the Mo_2_C catalyst differed from when using a palladium catalyst, due to different acyl-to-alkyl rearrangement on the surfaces. McManus and Vohs [14] investigated the deoxygenation of glycoldehyde and furfural with a Mo_2_C catalyst on a Mo (100) support. The catalyst was prepared as per the method described by Farkas and Solymosi [15] by which an orthorhombic structure of the molybdenum carbide is obtained with a molybdenum like facet. They observed an initial interaction with the surface through the aldehyde groups, and also noticed the weakening of the carbonyl C—O bond at temperatures of 200−300 K, which facilitates its dissociation. Bhan et al. also extensively studied the appropriateness of molybdenum carbide towards the HDO of hydrocarbons [[16], [17], [18], [19], [20]]. For the HDO of acetone over acidified molybdenum carbide [20], sequential hydrogenation led to formation of isopropyl alcohol, which was then followed by dehydration to form propylene and finally propane. For a similar investigation on the HDO of gas-phase anisole over a Mo_2_C catalyst surface [19], desirable high selectivity and efficiency was observed towards the cleavage of C—O bond, and high stability of the catalyst, at conditions of 420−520 K and 1 atm of pressure.

To understand the fundamental aspects of the reaction mechanism, computational studies provide important insights. Shi et al. [21] investigated, using density functional theory (DFT), the Mo_2_C (101) surface for catalytic conversion of butyric acid to butane, considering the reaction as representative of conversions of fatty acids to long-chain alkanes. The highest energy barrier in the reaction profile is observed for the dissociation of butanol. In complementary calculations, they also showed that the Mo_2_C (001) surface was able to adsorb water, OH, and O, more strongly than the (101) surface. Shi et al. [22] also investigated the conversion of furfural to 2-methylfuran on the Mo_2_C (101) surface, comparing bare surfaces with those pre-saturated with H atoms. At the elevated hydrogen coverage, the selectivity of the products could be tuned: at “high” H_2_ pressure, the formation of 2-methylfuran was promoted whereas the furan formation was suppressed. Luo et al. [23] studied formic acid decomposition over *β*-Mo_2_C (101) surface using DFT and report the cleavage of HCO—OH to be thermochemically most favourable route for formic acid decomposition.

Whilst the highlighted research provides clear evidence of Mo_2_C being a promising HDO catalyst, much of the mechanism of HDO remains unexplored on this material. Modern computational approaches, specifically electronic structure theory, provide an opportunity to interrogate mechanisms and identify the source of favourable catalytic properties in a material; therefore, herein the dehydration and dehydrogenation of formic acid on the molybdenum carbide surface has been investigated. Formic acid is an ideal model compound to simulate the HDO process because it is the smallest carboxylic acid present in bio-oil, and thus, the results can provide understanding that can be generalised to the decomposition of carboxylic acids on Mo_2_C surfaces. Furthermore, formic acid is a potential liquid hydrogen carrier *in situ* for fuel cells and hydrogen powered engines, making it desirable to understand its molecular properties and conversion pathways on promising catalysts [[24], [25], [26], [27]]. Complementary DFT and microkinetic calculations are outlined herein (Section 3) and used to study the energetics of the conversion pathways and determination of kinetic parameters (Section 4).

## 2 Reaction scheme

The decomposition of formic acid proceeds mainly via dehydration or dehydrogenation [28]. In the dehydration mechanism, formic acid decomposes to CO and H_2_O, whereas in the dehydrogenation mechanism, CO_2_ and H_2_ are formed. A third mechanism is also reported for decomposition to formaldehyde (HCHO), which is observed in trace quantities in non-catalytic reactions [29]; however, since such a mechanism is not reported in catalytic investigations, the formaldehyde product has not been considered further.

**Fig. 1:**
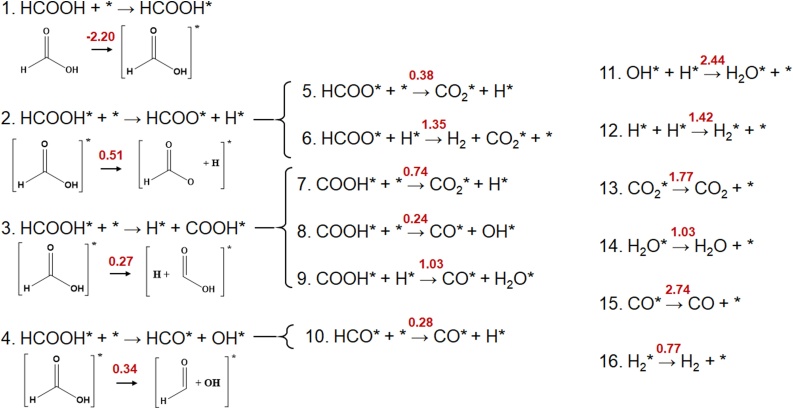
Complete reaction scheme, showing all the reactions considered in this study for the dehydration and dehydrogenation of HCOOH. The energy barrier (in eV) for each reaction, as calculated in this work, is presented in red (For interpretation of the references to colour in this figure legend, the reader is referred to the web version of this article.).

All the elementary reactions considered for the dehydration and dehydrogenation mechanisms are shown in Fig. 1. For ease of understanding, the reactants, products and transition states are herein referred to as *X*r, *X*p and *X*TS, respectively, where *X* is the reaction number in Fig. 1. The surface site is represented by *, with an adsorbed species denoted by their chemical formula followed by *.

The reaction scheme begins with the adsorption of formic acid (1r) on to the surface to give 1p. As the cleavage of the C=O bond is energetically demanding (7.67 eV) [30,31] and therefore, low probability, the adsorbed formic acid (HCOOH*) can begin to break down in three ways:

1 The H atom can break from the hydroxyl group to give HCOO* and H* (Reaction 2);
2 Alternatively, the C—H bond can break to give H* and COOH* (Reaction 3);
3 The entire hydroxyl group can break apart from the C atom to give HCO* and OH* (Reaction 4) [30,31].

The HCOO* formed in reaction 2 can dehydrogenate in two ways to give CO_2_: in reaction 5, the HCOO* breaks down to give CO_2_* and H*; alternatively, the HCOO* can break down to H* and CO_2_*, and the H* then reacts with another H* on the surface to form H_2_ in an associative desorption process; thus, the final products are the adsorbed CO_2_* and gas-phase H_2_. Similarly, the COOH* formed in reaction 3 can produce CO_2_, CO and H_2_O in reaction 7, 8 and 9. In reaction 7, the H from the OH group of COOH* cleaves to form CO_2_* and H*, while in reaction 8 the OH group cleaves from COOH* to form CO*; and in reaction 9, the COOH* reacts with H* to form carbon dioxide and water on the surface. Finally, the HCO formed in reaction 4 can be degraded on the surface in reaction 10 to give CO*.

To maintain an overall closed reaction cycle, further reactions are also included for the formation of small molecules. Reaction 11 and 12 describe the formation of water and hydrogen from the pseudo-stable moieties present on the surface, and all the formed molecules desorb from the surface in reactions 13–16 (CO_2_, H_2_O, CO and H_2_, respectively).

## 3 Methodology

### 3.1 Bulk

Density functional theory (DFT) simulations were performed using the “*Fritz Haber Institute ab initio molecular simulations*” (FHI-aims) software package [32], coupled with the “*Atomic Simulation Environment*” (ASE) Python package [33] for the management of model geometries.

**Fig. 2:**
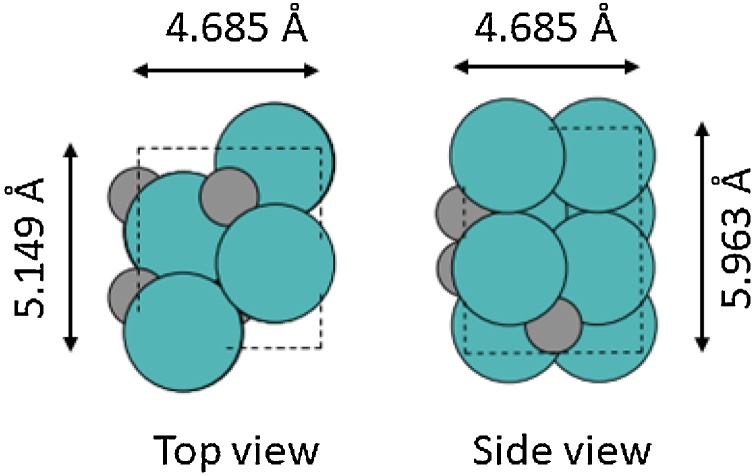
Top view and side view of the bulk *β*-Mo_2_C with unit cell lengths, where Mo atoms are represented by teal colour, carbon atoms by grey colour and the dashed line show the unit cell (For interpretation of the references to colour in this figure legend, the reader is referred to the web version of this article.).

The orthorhombic (*β*) structure of Mo_2_C (Fig. 2) is stable at a large range of temperatures [34], and has been considered as the catalytic form of the material during our investigation. A converged 5 × 5 × 4 **k**-grid was chosen for the periodic bulk calculations as, when performing convergence testing for the **k**-grid density, subsequent increments in **k**-grid sampling altered total energies by < 10 meV (1 kJ mol^−1^) (Table 1S). Relativistic effects were included using zeroth order regular approximation (ZORA) [32] as a scalar correction, and the spin and charge for the system were set to zero. Default FHI-aims convergence criteria was used for the self-consistent field (SCF) energy calculations, such that changes in the charge density (Δ*ρ*) for the *N* atom system were:

(1) Δ*ρ* ≤ 10^−6^ x (*N* / 6) *e* a_0_^-3^

To ensure energetic accuracy in a computationally efficient manner, the available basis sets of the FHI-aims package [32] were compared; the *light* basis set, which is a double-numerical (DN) with polarization basis set, was deemed appropriate as it provided converged accuracy in a tractable timeframe.

A range of exchange-correlation (XC) density functionals were compared for appropriateness towards modelling *β*-Mo_2_C. For each XC, the bulk model was optimized using the trust-region method [35] until the force on each atom was less than 0.01 eV Å^−1^; the unit cell vectors were also optimized, with the angles between the lattice vectors fixed (i.e. orthorhombic symmetry preserved). Comparison of the cohesive energy, formation energy, and unit cell parameters with literature for *β*-Mo_2_C shows the PBE [36] XC functional with the Tkatchenko-Scheffler van der Waals correction [37] as providing favourable accuracy as well as computational efficiency (Table 2S); more extensive and computationally exhaustive functionals did not improve the target observables significantly. Thus, the PBE+TS XC functional is used for the continuation of this study.

### 3.2 Surfaces

**Fig. 3:**
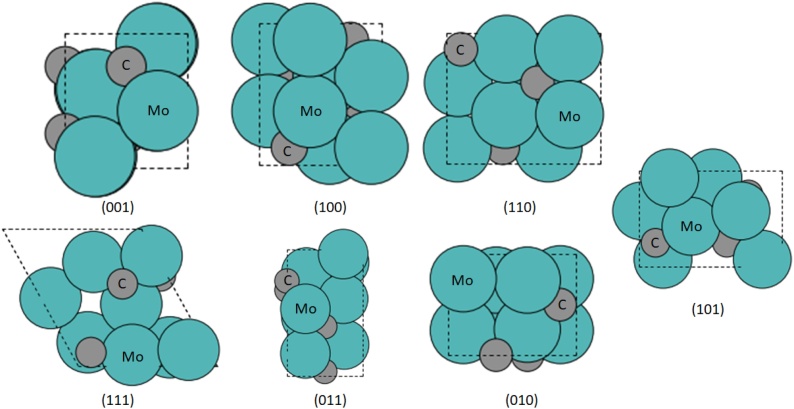
Top view (i.e. *xy* plane) of the investigated facets of *β*-Mo_2_C with Mo terminations, where teal and grey circles represent molybdenum and carbon atoms, respectively, and the unit cell is represented by the dashed lines (For interpretation of the references to colour in this figure legend, the reader is referred to the web version of this article.).

Seven different low-index vicinal surface facets (Fig. 3) were created from the optimized bulk unit cell of *β*- Mo_2_C: namely the (100), (110), (111), (101), (010), (001), and (011) facets. The Mo-terminated facets are reported to exhibit similar reactivity as that of Ru, Ni, and Pd metals [38]; in particular, the dissociation energy for a C—C bond on the *β*- Mo_2_C surface is similar, and the dissociation of the C—O bond is thermodynamically more favourable. The carbon terminations are also known to reduce the C—O bond scission [39]. Therefore, only the metal terminations for the facets are considered herein. Furthermore, recent experimental and computational studies report that the (100) facet is highly active for hydrodeoxygenation (HDO) reactions [40,41]; the same surface has a high affinity towards hydrogen adsorption [42], which is a favourable characteristic for an HDO catalyst [43]. Furthermore, the Mo terminated (100) facet has the highest Mo surface density (0.130 atom Å^−2^), providing higher coordination sites and resulting in better catalytic activity [44]. Experimentally, the surface can be synthesized using the chemical vapour deposition technique [45], or through direct carburization of molybdenum [15] or molybdenum oxide [46]. Therefore, it is important to identify the relative stability of this surface, as it is used in all later work.

**Fig. 4:**
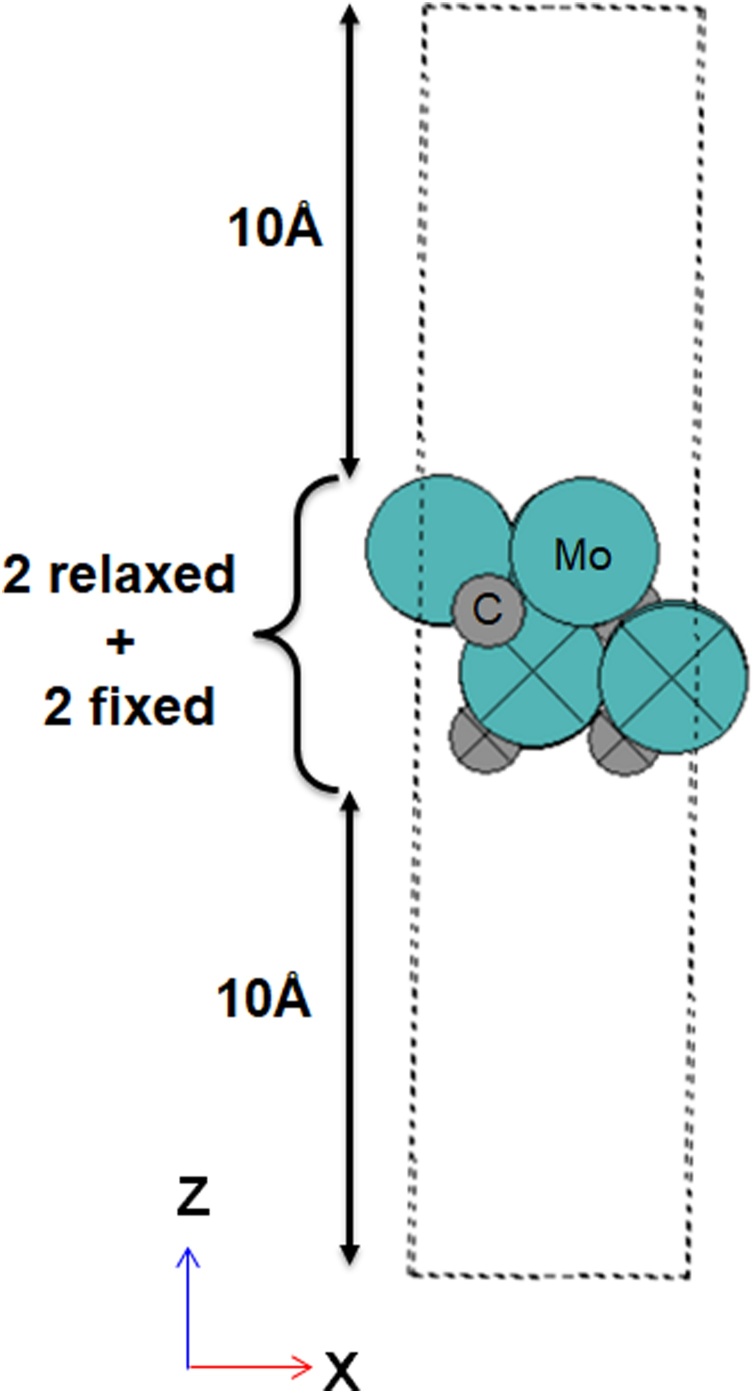
Profile of the (100) slab model, in the *xz*-plane. Colours are as per Fig. 3, with fixed atoms noted with crosses. The 10 Å vacuum above and below the slab (i.e. 20 Å total) is also presented.

A 10 Å vacuum was introduced perpendicular to the *xy* plane both above and below the surface, meaning that slab surfaces where separated by 20 Å in total (Fig. 4). The large vacuum region eliminates self-interaction between periodic slab models. In order to determine the relative stability of facets, the surface energy $\left(\gamma_{hkl}\right)$ of each individual face, with miller indices (*hkl*), is calculated for a single-sided four-layer slab as:

(4) $$\gamma_{hkl}=\frac{1}{A}\left[E_{\text{M}{\text{o}}_{2}\text{C}}^{\text{s}\text{u}\text{r}\text{f,rel}}-\frac{s∙E_{{\text{Mo}}_{2}\text{C}}^{\text{bulk}}}{2}-\frac{E_{{\text{Mo}}_{2}\text{C}}^{\mathrm{surf},\mathrm{fix}}}{2}\right]$$

where $E_{\text{M}{\text{o}}_{2}\text{C}}^{\text{s}\text{u}\text{r}\text{f,fix}}$ and $E_{\text{M}{\text{o}}_{2}\text{C}}^{\mathrm{surf},\mathrm{rel}}$ are the total energies of the surface model in the bulk structure and after optimisation, respectively, $E_{\text{M}{\text{o}}_{2}\text{C}}^{bulk}$ is the total energy of the bulk unit cell, respectively, *s* is the number of bulk units in the slab model, and *A* is the surface area in the *xy* plane on the top of the slab. For the study of chemical reactions, a one-sided model of adsorption is favourable because it minimises calculation complexity. To compensate for the inhomogeneous electric field that arises in such one-sided calculations, a dipole correction is applied.

For the (2 x 2) surface facets, the lowest surface energy is calculated for γ_111_ (3.43 J m^−2^) and the highest surface energy is calculated for γ_100_ (4.54 J m^−2^, Fig. 1S), with a total range of 1.11 J m^−2^. Whilst not the *most* energetically favourable [43], the Mo-terminated (100) facet is relatively stable and, therefore, investigated in the continuation of this study. To determine a chemically accurate thickness for the (100) slab model, γ_100_ was calculated as a function of slab thickness, with the bottom half of the slab atoms fixed in position; γ_100_ differs by 1.04 J m^−2^ between two and four layers, and then by less than 0.14 J m^−2^, when comparing four, six and eight layers; thus, for a balance of computational efficiency and accuracy, subsequent calculations are performed with a four-layer model. Furthermore, the constraints applied to ensure bulk structure at long-range from the adsorption site, i.e. on the side of the slab without an adsorbate, were validated through impact on γ_100_; for a model with four layers, constraints to the lower two layers alter γ_100_ by only 0.25 J m^−2^ compared to the fully constrained slab (Table 3S), and these constraints are applied herein.

Calculations were performed of the adsorption, reaction and desorption of the molecules, as outlined in the reaction scheme in Fig. 1. The adsorption energy ($E_{\text{a}\text{d}\text{s}}$) is calculated as:

(5) $$E_{\text{a}\text{d}\text{s}}=E_{\text{M}{\text{o}}_{2}\text{C}+\text{M}\text{o}\text{l}\text{e}\text{c}\text{u}\text{l}\text{e}}-E_{\text{M}{\text{o}}_{2}\text{C}}^{\text{s}\text{u}\text{r}\text{f,rel}}-E_{\text{M}\text{o}\text{l}\text{e}\text{c}\text{u}\text{l}\text{e}}$$

where $E_{\text{M}{\text{o}}_{2}\text{C}}^{\text{s}\text{u}\text{r}\text{f,rel}}$ is the energy of the surface, $E_{Molecule}$ is the energy of the adsorbate and $E_{{\text{M}\text{o}}_{2}\text{C}+\text{M}\text{o}\text{l}\text{e}\text{c}\text{u}\text{l}\text{e}}$ is the energy of the combined system, all in optimised geometries. As with any thermodynamic process, the reverse action (i.e. desorption, *E*_des_) can be calculated as *E*_des_ = - *E*_ads_.

The adsorption energy of all the molecules was calculated at varying orientations on the different available surface sites: *on-top*, *bridge*, *fcc* and *hcp*. Two *hcp* sites (Mo-*hcp* and C-*hcp*) are considered depending on the type of atom present underneath the top layer. The most stable arrangement (i.e. most negative *E*_ads_) are considered for the full reaction pathways, with the transition states calculated using a climbing image nudged elastic band (CI-NEB) method [47] with the molecular dynamics based Fast Inertial Relaxation Engine (FIRE) optimization algorithm [48]. A minimum of seven images (including reactants and products) were used in the transition state search, and the convergence criteria for the force on atoms was set to 0.06 eV Å^−1^. To confirm the stability of individual minima and transition states, vibrational frequencies were calculated; the presence of exactly one imaginary frequency along the reaction pathway confirmed that transition states were first order saddle points on the potential energy landscape [49].

### 3.3 Microkinetics

Thermochemical analysis and microkinetic modelling of the reactions [50,51] was performed to determine the system behaviour [52] using our in-house modelling code [53]. The global partition function was calculated as *Q* = *q_vibrational_* **·***q_rotational_* **·** *q_translational_* **·***q_electronic_*. The vibrational frequencies for each model were used to calculate the vibrational partition function (*q_vibrational_*). The rotational (*q_rotational_*) and translational (*q_translational_*) contributions to *Q* were derived from the molecular structures and assume to be unity for adsorbed species as rotations and translations are frustrated. The electronic (*q_electronic_*) partition function was set as the electron multiplicity in the ground state and assuming no excitations in the working temperature range. Subsequently, thermodynamics parameters (i.e. enthalpy, *H*, and entropy, *S*) were calculated at different temperatures (*T*) assuming the harmonic approach [52] and with the following relations:

(6) $$S=k_{B}\;\text{ln}\;Q+kT{\left(\frac{\partial ln\;Q}{\partial T}\right)}_{\text{V}}$$

(7) $$C_{p}=T{\left[\frac{\partial S}{\partial T}\right]}_{\text{p}}$$

(8) $$H=E_{DFT}+E_{ZPE}+\int_{0}^{T}C_{\text{p}}\partial T$$

(9) *G* = *H* – *TS*

where *k*_B_ is the Boltzmann constant, *V* is volume, *p* is pressure, *E*_DFT_ is the calculated DFT electronic energy, *E*_ZPE_ is the vibrational energy correction at 0 K, *C*_p_ is the heat capacity at constant pressure, and *G* is the Gibbs free energy. To validate our approach, the thermochemical parameters for the gas phase species were compared with literature [54,55], with all values in very good agreement (Table 4S in the SI). The maximum difference in the *C*_p_ and *S* for the studied molecules are less than 7.65 J mol^−1^ K^−1^ in the studied temperature range, and the maximum difference in the enthalpy values is less than 3.62 kJ mol^−1^ (Table 4S).

The rate constant (*k*) of each of the elementary reaction was calculated using the transition-state theory (TST) approximation of Eyring, Evans and Polanyi [56,57]. For this, the calculations were carried out using the relation:

(10) $$k=A_{0}\;exp\left(\frac{-\Delta G^{\neq }}{k_{\text{B}}T}\right)=\frac{k_{\text{B}}T}{h}\frac{Q_{\text{TS}}}{Q_{\text{r}}}\;\exp\left(\frac{-\Delta G^{\neq }}{k_{\text{B}}T}\right)$$

where *A*_0_ is the pre-exponential factor, $\Delta G^{\neq }$ is the activation free energy of the reaction, and *Q*_TS_ and *Q*_r_ are the partition functions of the transition state and reactant, respectively. Adsorption and desorption processes from the surface were considered barrier-less, but maintaining consideration of the variable degrees of freedom, e.g. 2D and 3D. Therefore, the adsorption rate constant (*k*_ads/des_) was calculated from the Hertz-Knudsen relation [58] as:

(11) $$k_{\text{ads/des}}=A_{0}S_{0}\left(T\right)=\frac{A_{\text{cat}}}{\sqrt{2\pi m_{i}k_{\text{B}}T}}S_{0}\left(T\right)$$

where *A*_cat_ is the surface area of catalyst, *i.e.* the area in the simulation cell, and the sticking coefficient *S*_0_(*T*) is a temperature dependent term calculated from 3D and 2D degrees of freedom from the global partition function. The energy barrier for the diffusion of molecules on the surface was considered as negligible. To develop the kinetic model, the rate constants for the adsorption process were considered as

(12) *r_i_* = *A*_0_*S*_0_(*T*)*P*_i_*θ*_i_

and the rate constants for the surface reactions and desorption processes considered as

(13) $$r_{i}=A_{0}exp\;\left(\frac{-\Delta G^{\neq }}{k_{\text{B}}T}\right)\theta_{i}$$

Here, *P*_i_ is the partial pressure of species *i*, and *θ*_i_ is the fractional surface coverage of the same species such that ∑*θ*_i_ = 1 throughout. The differential equations were formulated as:

(14) $$\frac{d{\text{χ}}_{i}}{dt}=r_{x_{i}}$$

where χ_i_ is *P*_i_ for a gas phase species or *θ*_i_ for a surface species, and $r_{x_{i}}$ is the net reaction rate for the formation of species *i*. Finally, the system of ordinary differential equations (ODEs) were solved to obtain a steady state solution for the system.

To complement the kinetic rate model, simulations of the temperature programmed desorption (TPD) technique were also considered. In this model, the temperature of the system was increased at the rate of 10 K s^−1^ and the surface was fractionally covered with hydrogen adatoms along with HCOOH. In line with experiments, where the gases are continuously removed from the reactor, re-adsorption of the evolved gases to the surface was not included in the ODEs. Overall, four different scenarios for initial HCOOH coverage on the surface were considered: 0.1, 0.4, 0.7 and 1.0 ML (monolayer).

## 4 Results and discussion

### 4.1 Reaction profiles

**Fig. 5:**
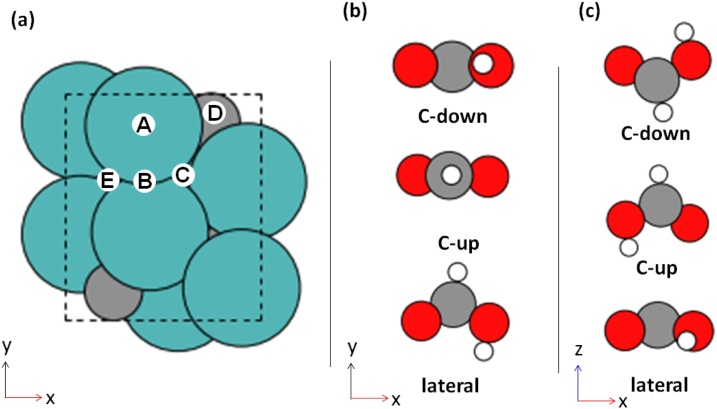
(a) Top view (*xy*-plane) of the catalyst surface where the possible adsorption sites are identified as: A, *atop*; B, *bridge*; C, Mo-*hcp*; D, C-*hcp* site; and E, *fcc*. Atom colours are as in Fig. 3. (b) Top view (*xy*-plane) and (c) side view (*xz*-plane) of HCOOH in different arrangements considered for adsorption. Oxygen, carbon and hydrogen atoms are shown in red, grey and white, respectively (For interpretation of the references to colour in this figure legend, the reader is referred to the web version of this article.).

The conversion of formic acid initiates with adsorption of the molecule on to the *β*-Mo_2_C surface. Five non-equivalent sites were examined to determine the most stable adsorption configuration of formic acid over the surface, namely the *atop*, *bridge*, Mo-*hcp*, C-*hcp* and *fcc* positions, and three different arrangements were considered for the molecule, namely *lateral*, *C*-*up* and C-*down*, as shown in Fig. 5(a).

**Fig. 6:**
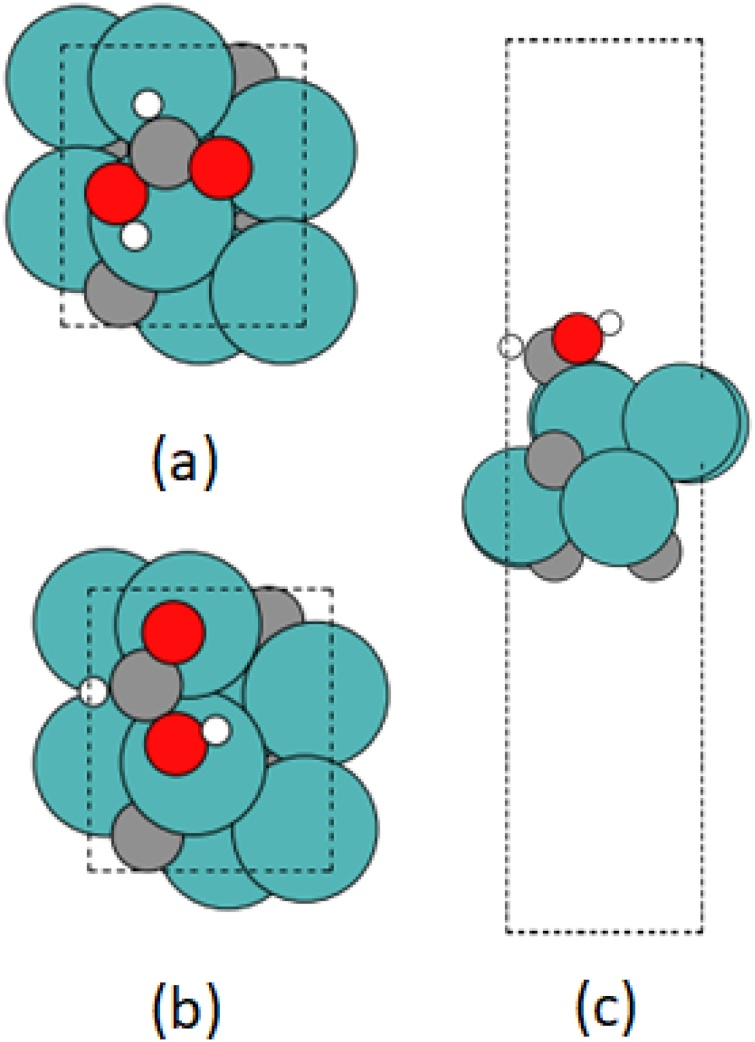
The configuration of HCOOH positioned *laterally* on the surface *bridge* site, which is deemed the most stable. (a) Initial geometry, viewed in the *xy*-plane; (b) optimized geometry, viewed in the *xy-* and (c) *xz-*plane. Atom colours are same as in Fig. 5.

The adsorption energies were calculated for each optimized configuration. The most stable adsorption of HCOOH occurs in the *lateral* configuration over the *bridge* site (Fig. 6), with an *E*_ads_ of −2.20 eV. On a pure transition metal, usually the C-*up* position is more stable as the oxygen atoms are able to bond with the metal surface with high coordination [59]; however, in carbides, the carbon atom of the adsorbate tends to shift towards the carbon deficit site [60]. Thus, during the geometry optimization, the molecule repositioned such that the carbon atom partially covered the *fcc* site and the oxygen atoms occupy the *atop* position. For the next most stable adsorption, which is with the molecule positioned *laterally* at the C-*hcp* site (*E*_ads_ = −2.11 eV), a similar relocation of the formic acid species occurs and the C atom again partially covers the Mo-*hcp* site. Initial and optimized structures are presented together in Figs. 2S and 3S. In general, the *lateral* configuration of HCOOH is the most stable for each adsorption site, with *E*_ads_ between −1.85 and −2.20 eV; furthermore, in several cases for the *C-up* and *C-down* configuration, the molecules optimise to a *lateral* configuration. Overall, the adsorption energy is considerably higher on *β*-Mo_2_C than on other metal surfaces [61], which we attribute to the interaction of at least 3 atoms (2 oxygen and 1 carbon atom) with the surface.

**Fig. 7:**
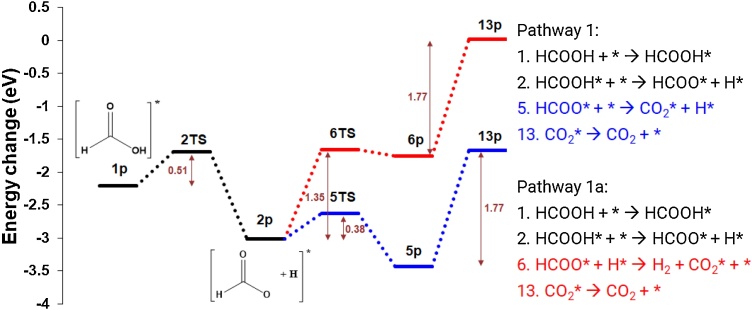
Potential energy surface (PES) of the dehydrogenation of HCOOH, via the formation of formate, to produce carbon dioxide via pathway 1a (blue) and 1b (red) (For interpretation of the references to colour in this figure legend, the reader is referred to the web version of this article.).

The conversion of formic acid has been considered via three different pathways: 1 (1a and 1b), 2 (2a, 2b and 2c) and 3a. In pathway 1 (Fig. 7), the HCOOH dehydrogenation proceeds by cleavage of the O—H bond to produce formate (HCOO*) and hydrogen (H*) on the surface, with an activation energy of 0.51 eV. The formate (5p) can break down further to form CO_2_* and H* (Pathway 1a, *E*_act_ = 0.38 eV), or react with H* to form CO_2_ on the surface and H_2_ in the gas phase (Pathway 1b, *E*_act_ = 1.35 eV). The greater activation energy for pathway 1b suggests dehydrogenation via pathway 1a is most likely. The adsorption of CO_2_ on the surface, calculated by systematically assessing the interaction of CO_2_ with the five different surface sites and in *lateral* and *vertical* orientations (Fig. 4S), identifies the most stable arrangement as the partial *bridge* site (*E*_ads_ = −1.77 eV); the *vertical* configuration over the *fcc* site also realigns to this configuration during optimization. Interestingly, and contrastingly, adsorption in the *atop*, *lateral* configuration leads to the dissociation to CO and O on the surface, with CO_2_ dissociative adsorption highly favourable (*E*_ads_ = −3.75 eV). The dissociation is unsurprising given Mo_2_C is reported to be selective towards the cleavage of carbon and oxygen bonds [61]. For pathways 1a and 1b, the final desorption is considered to proceed as associated CO_2_, with an *E*_act_ of 1.77 eV.

**Fig. 8:**
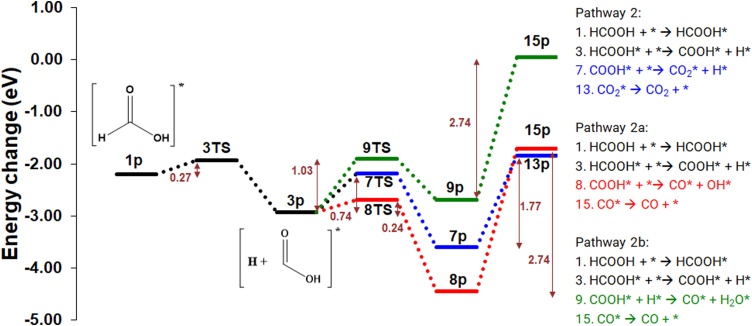
Potential energy surface (PES) for the dehydration and dehydrogenation of HCOOH on the catalyst surface to produce CO and CO2 in pathway 2a (blue), 2b (red) and 2c (green) (For interpretation of the references to colour in this figure legend, the reader is referred to the web version of this article.).

The second dissociative pathway (2) also commences with the adsorption of HCOOH (*E*_ads_= −2.20 eV) and cleavage of the H—COOH bond, leading to the formation of co-adsorbed COOH* and H*. *E*_act_ for the breaking of the H—C bond is 0.27 eV, while the reaction energy is −0.76 eV, leading to the formation of 3p via a thermodynamically driven step. The COOH* can break down subsequently to CO_2_ and CO in three different ways. In pathway 2a, the COOH* breaks down onto CO_2_* and H* (*E*_act_ = 0.74 eV), with the CO_2_* then desorbed as described in pathway 1a (Fig. 7, Fig. 8).

Alternatively, in pathway 2b, the COOH* dehydrates to CO* and OH* (*E*_act_ =0.24 eV), which is lower than the reported activation energy on other precious metal surfaces: on the Au (100) surface, *E*_act_ is 0.70 eV [28]; on the Pd (111) surface, *E*_act_ = 1.20 eV [62]. On the Pt (111) and (100) surfaces, the reaction occurs via the formation of cis—COOH with *E*_act_ of 0.61 eV and 0.57 eV [63], respectively. In our results, the consequential reaction energy for the step is exothermic (−1.51 eV), which is again more favourable than on precious metal surfaces such as Au (100) [28], Pd (111) and Pt (111) [62]. For the CO molecule on the surface, a systematic study was conducted with two different configurations (*C-up* and *C-down*) on the five different sites previously identified (Fig. 5S). In general, the CO stabilises at carbon deficient sites with a strong adsorption energy (*E*_ads_ < −0.60 eV), in agreement with Nagai et al. [60]. The most stable CO adsorption is the *bridge*, *C-down* configuration but partially covering the *fcc* site (*E*_ads_ = -−2.74 eV); Thus, the CO desorption energy is considered herein as requiring 2.74 eV (8p→15p). The high desorption energy makes the *β*-Mo_2_C (100) surface highly susceptible to CO poisoning, which is previously observed [64].

In pathway 2c, COOH* reacts with H* via 9TS to give CO and H_2_O on the surface. The activation energy for this reaction is 1.03 eV, which is the most energy demanding pathway calculated for CO production. As discussed above, the CO desorption energy is high (9p→15p, 2.74 eV) and would be the rate determining step in this reaction pathway; and the surface would be susceptible to poisoning.

**Fig. 9:**
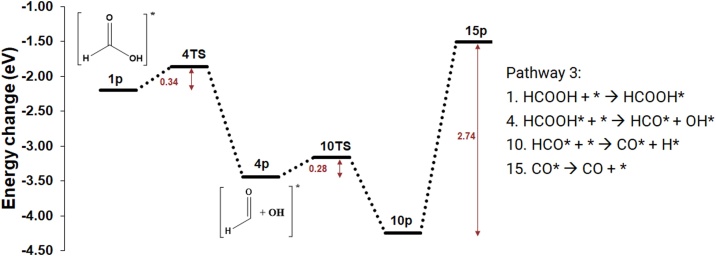
Potential energy surface (PES) for the most favourable pathway for the dehydration of HCOOH producing CO in pathway 3a.

In a third pathway, the HCO—OH bond is cleaved in reaction 4, as shown in pathway 3a of Fig. 9. The transition state is 0.34 eV above reactants, making it the next most favourable conversion route after reaction 2. Further, the HCO* is broken down into CO* and H* on the surface in reaction 10, which is facile (*E*_act_ = 0.28 eV) and can be driven by the exothermic reaction energy (−0.81 eV). As already highlighted, the subsequent desorption of CO is a very endothermic process.

In several of the presented reaction schemes, H* and OH* form and can react to give hydrogen or water molecules on the surface (Reaction 11 and 12, respectively, in Fig. 1). The formation of H_2_O* proceeds via transition state 11TS with *E*_act_ = 2.44 eV, whereas H_2_* proceeds via 12TS with *E*_act_ = 1.42 eV. The H_2_ stability on the surface is reportedly unaffected by the carburization of molybdenum carbide [65], with other transition metal carbides (TiC, VC, ZrC and NbC) favouring dissociation of hydrogen on the surface [66]. Here, *β*-Mo_2_C exhibits similar behaviour, with only dissociative adsorption of H_2_ observed (*E*_ads_ = −0.77 eV), i.e. the H_2_ molecule is unstable, with the separated H atoms positioned on the *atop* position, in agreement with earlier reports [62]. The stability of the water molecule was confirmed by considering all adsorption sites and *O-up* and *O-down* configurations (Fig. 6S); The most favourable position is clearly in the *atop lateral* position (*E*_ads_ = −1.03 eV), as all models converge to this configuration.

**Fig. 10:**
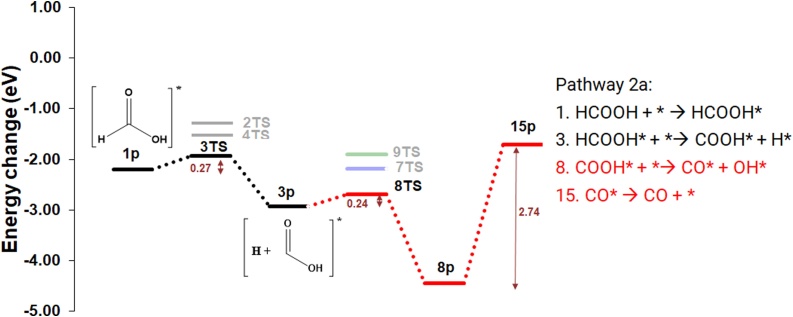
Most favourable pathway (2b, red) for the conversion of formic acid on *β*-Mo_2_C surface. The crucial competing step that determines selectivity to CO_2_ or CO is shown with alternative paths given in blue (pathway 2a) and green (pathway (2c) (For interpretation of the references to colour in this figure legend, the reader is referred to the web version of this article.).

Overall, the most suitable pathway for the conversion of the formic acid is via reaction 3 to form COOH* on the surface, with all the optimized molecular geometries of all the reactants, transition states and products are given in Figs. 7S and 8S. The reaction requires 0.27 eV as initial activation energy, and subsequently formed COOH* degrades to CO* and OH* (via 8TS*) with an activation energy of 0.24 eV. Whilst the final CO desorption from the surface requires 2.74 eV (Fig. 10), the feasibility of this step is considered below with microkinetic modelling. Ideally, conditions would be sought that promote reaction 7, leading to CO_2_ formation, over reaction 8, as the CO surface bond is strong; alternatively, the surface could be modified such that the CO desorption energy is reduced, and the active site recycled, though this approach is not considered further here.

### 4.2 Microkinetics modelling

#### 4.2.1 Batch reactor model

A batch reactor model with an initial HCOOH pressure:surface ratio of unity was considered in the temperature range of 300–500 K. All elementary steps listed in Table 5S were considered in the microkinetics simulations; at each temperature, the concentration of each species was calculated during the first second of the reaction, i.e. time period from 0 to 1 s in steps of 0.01 s. under the assumption that steady-state is reached within this time. For the adsorption reactions, the temperature dependent sticking coefficient (*S*_0_(*T*)) was also calculated (Table 5S).

**Fig. 11:**
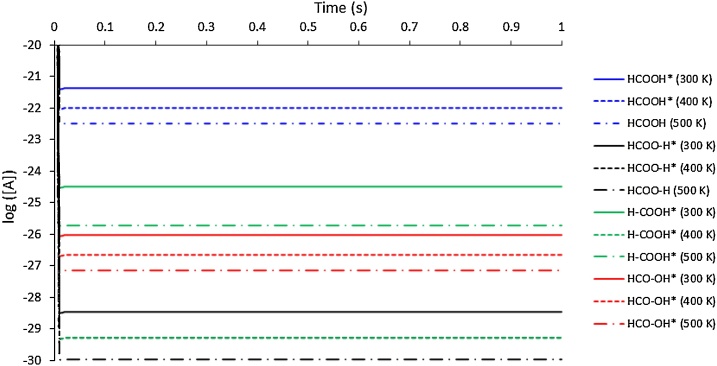
Plot of log of steady-state concentration [A] of 1p (HCOOH*), 2p (HCOO-H*), 3p (H-COOH*—) and 4p(HCO-OH*) on the surface with time at 300 K, 400 K and 500 K.

The rate constant (*k*) for the HCOOH adsorption process (reaction 1) is very small (of the order 10^−4^ s^−1^), and therefore the concentration of formic acid on the surface is tiny (of the order 10^−22^ ML (monolayer) at 300 K) and remains steady (Fig. 11). With an increase in temperature, the adsorption rate decreases for HCOOH and its concentration drops further as conversion increases. At 400 K and 500 K, the HCOOH* concentration at steady-state is of the order 10^−23^ ML and the surface is largely vacant, which agrees with the simulation model of low coverage. Since all the products are a result of the breakdown of HCOOH, the concentration of all the species is proportional to HCOOH*. The steady-state concentration of species 2p, 3p and 4p suggest that the most favourable mechanism of the HCOOH decay is reaction 3 followed by reaction 4, and finally reaction 2. As the temperature is increased from 300 K to 400 K, the steady-state concentration of 4p is steady compared to the change in the concentration of 2p and 3p, both of which decrease by an order of magnitude, which indicates that the path leading to 4p is mostly unaffected at this range of temperatures. However, from 400 K to 500 K, the trends reverse, i.e. the change in the concentration of 2p and 3p is minimal while the concentration of 4p changes by 10^−1^ ML. This dramatic change is because the backward reaction rate for reaction 4 increases from 400 K, and therefore the concentration of 4p decreases.

**Fig. 12:**
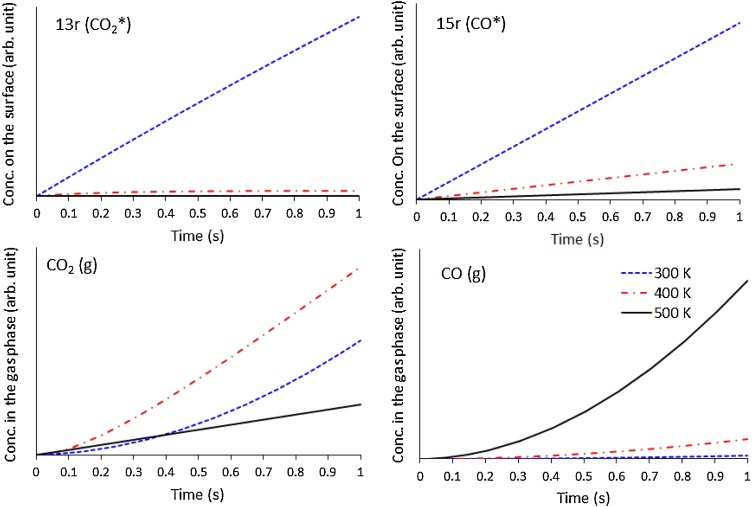
Plot of steady-state concentration of 13 r and 15 r on the surface, and CO and CO_2_ in the gas phase, against time, at 300 K, 400 K and 500 K. The plots are showing the ratio between these gaseous species and the initial HCOOH, and are normalised by: 10^−13^ for 13 r, 10^−11^ for 15 r, 10^−14^ for CO_2_(g) and 10^−22^ for CO(g).

Further degradation of 2p (HCOO-H*), 3p (H—COOH*) and 4p (HCO—H*) to form CO and CO_2_ species on the surface occurs as shown in Fig. 12. At 300 K, the concentration of CO_2_ on the surface rise steadily as the time progresses. This is due to the continuous formation of CO_2_ on the surface from 2p and 3p via reaction 5, 6 and 7. As the surface concentration of CO_2_ increases, desorption of CO_2_ from the surface into the gas phase also increases. At elevated temperatures (i.e. 400 K and 500 K), the formation of CO_2_ on the surface decreases, which is attributed to two factors: (i) the formation of 2p and 3p decrease, and therefore there is less reactant for the formation of CO_2_, and (ii), the rate constants of reaction 5 and 7 also decrease with increase in temperature (Table 5S). Thereafter, whatever CO_2_ is formed on the surface at 400 K and 500 K is desorbed almost immediately due to the high rate constant of reaction 13.

From 3p and 4p, carbon monoxide is formed on the surface *via* reactions 8, 9 and 10, and accumulates on the surface at all the temperatures studied. At 300 K, 100 times more CO is produced on the surface than CO_2_, and the *k* for CO formation increases faster with the temperature than for CO_2_. These, together with the very slow CO desorption, lead to a difference in the CO and CO_2_ coverage of 10^5^ at 500 K, presenting a ratio of 10^−22^ with the initial HCOOH at all temperatures. The slow desorption kinetics (2.33 × 10^−11^ s^−1^at 300 K) of CO suggests that it could poison the surface at low to moderate temperatures.

#### 4.2.2 Temperature programmed desorption (TPD) model

In order to see how HCOOH coverage affects the reactions, TPD modelling was conducted. Four different scenarios were considered with increasing initial formic acid surface coverage: 10 %, 40 %, 70 % and 100 %. The temperature was increased at the rate of 10 K s^−1^ and the change in the concentration of all the gas phase species was calculated with increasing temperature.

**Fig. 13:**
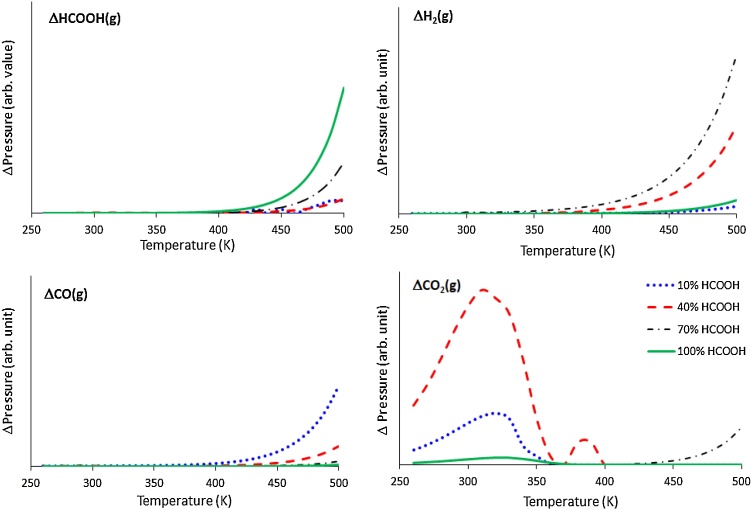
Plot of change in ratio of gaseous species (HCOOH, H_2_, CO_2_ and CO), with increasing temperature, for the different initial formic acid surface coverage.

Formic acid starts to appear in the gas phase after 400 K at all the four initial conditions (Fig. 13). The change in CO_2_ concentration begins at 260 K, reaches a peak at 340 K and falls thereafter for 10 % and 40 % HCOOH coverage. A tiny peak for CO_2_ is also observed at 100 % coverage conditions between 300−350 K; however, as vacant sites are not available for the reaction to occur, the peak is very small. The experimental TPD also reports the CO_2_ desorption near this temperature for different transition metal at similar coverage [67,68]. On the Pd surface, CO_2_ desorption from Zn is reported between 300 K and 400 K for 10%–50% concentration [67]. Similarly, CO_2_ is reported to evolve at 295 K over oxygen exposed Pd (110) [68]. The evolution of CO_2_ from our system suggests that the adsorbed formic acid is undergoing dehydrogenation at 260 K to 340 K through pathway 1. Above 400 K, CO is observed in gas phase indicating pathways 2 and 3 dominate the conversion route. The presence of CO at this temperature is in line with the experimental observations for other metals [67,69]. In the work of Flaherty et al. [39], the peak is observed before 400 K for CO over carbide of molybdenum; it is noted though that the species concentration in their case on the surface was 1.6 ML, and therefore the molecules from the second layer could be desorbing into the gas phase at lower temperatures than 400 K. On a Zn loaded Pd surface, CO appears in the gas phase between 300 K and 520 K, depending on the amount of Zn loading [67]. Similarly, over a CeO_2_ catalyst, CO appears from 450 K and peaks at 600 K [69]. Contrary to what is generally observed for metal catalysts [67,68], the change in the concentration of H_2_ is not observed before 350 K. For metal catalysts, the electronegative hydrogen adatoms are stabilised by the electropositive metals as they move away from the surface [70] for associative desorption, but the presence of carbon in the Mo lattice in Mo_2_C decreases the overall electropositivity of the surface and increases the work function [71,72]; therefore, a higher temperature is required to facilitate the hydrogen atoms’ associative desorption from the surface. The formation of H_2_ on the surface (reaction 12) also has slow kinetics (10^−1^ s^−1^). At higher HCOOH coverage (70 % and 100 %), since very less or no sites are available for the conversion to proceed, no change in the concentration of CO and H_2_ is observed in the gas phase. Only after 400 K, when HCOOH desorbs from the surface, does the concentration of CO, CO_2_ and H_2_ begin to change in increasing proportion. The rate of change of HCOOH in the gas phase increases sharply after 450 K, and is steeper for 70 % and 100 % coverage than for 10 % and 40 % coverage.

The selectivity analysis of the system shows that CO_2_ is the preferred end product obtained in the gas phase, with almost 100 % selectivity, which is very close to the reported experimental result of over 96 % for the *β*-Mo_2_C [73].

## 5 Conclusion

The dehydration and dehydrogenation of formic acid is studied on the *β*-Mo_2_C catalyst surface using density functional theory. Different orientations of formic acid, CO, CO_2_ and H_2_O are adsorbed on the surface at different surface sites to determine the most stable orientation and site for each molecule. The most stable structures are used to study the dehydration and dehydrogenation pathways of the conversion and the thermochemistry is analysed. Thermodynamically, the conversion of the HCOOH is most likely to proceed by the breaking of H—COOH bond to yield CO as the end product (dehydration); however, the desorption energy for CO from the surface is high (2.74 eV), which suggests that the surface is susceptible to CO poisoning.

Microkinetic analyses were also conducted for the batch reactor model and product distributions were calculated up to for 1 s between 300–500 K. The adsorption kinetics of formic acid were observed as very slow over the surface. As a result, most of the surface sites were vacant and the conversion inefficient. The concentration profile shows that the appearance of gas-phase CO_2_ will be kinetically faster than gas-phase CO. The formation of CO will saturate the surface as the CO desorption step is very slow, and therefore rate limiting. TPD analysis is also conducted to determine the concentration of different gas species in the system with increasing temperature and increasing concentration of adsorbed formic acid. At 10 % and 40 % formic acid coverage, HCOOH conversion occurs at low temperatures; CO_2_ desorption is most favourable at 320 K, reaching a steady state at 370 K. All other species start desorbing from the surface after 400 K.

## CRediT authorship contribution statement

KA performed the calculations presented in this work. KA, NK and AL contributed to the conceptualization of the project. KA, AR and AL contributed to software choices and method development in this work. All authors contributed to the analysis of results and preparation of manuscript.

## Declaration of Competing Interest

The authors declare that they have no known competing financial interests or personal relationships that could have appeared to influence the work reported in this paper.

## Appendix A Supplementary data

The following is Supplementary data to this article: Additional information is provided in a separate section containing a more detailed discussion of the parameter testing for the computational simulations, the optimised configurations observed for all intermediates considered in the simulations, and the data derived from the microkinetic simulations.
