# Supplementary material for: Dehydrogenation and dehydration of formic acid over orthorhombic molybdenum carbide
Source: Catal Today. 2022 Feb 15;384-386:197–208. doi: 10.1016/j.cattod.2021.04.011 (PMC9380418; doi:10.1016/j.cattod.2021.04.011)
Supplement: Supplementary file 1 [file mmc1.pdf]

Dehydrogenation and Dehydration of Formic Acid over Orthorhombic Molybdenum Carbide

by

Kushagra Agrawal[1,2], Alberto Roldan[2] , Nanda Kishore[1] and Andrew Logsdail[2,*]

[1]Department of Chemical Engineering, Indian Institute of Technology Guwahati, Guwahati – 781039, Assam, India

[2]Cardiff Catalysis Institute, School of Chemistry, Cardiff University, Park Place, Cardiff CF10 3AT, Wales, UK

*Email: LogsdailA@cardiff.ac.uk

Contents:

Table 1S: Convergence testing of **k**-grid sampling. The energy change (ΔE) as a function of sampling is obtained for a unit cell of x = 4.754 Å, y=5.241 Å and z= 6.076 Å, with the selected converged settings highlighted in grey……………………………..…………………………3

Table 2S: Comparison of the formation energy (E_{form}), cohesive energy (E_{coh}), bulk volume and lattice parameters calculated in this work for different functionals (PBE [1,2], M06-L [3], PBE0 [4,5] and B3LYP [6]) (*light* basis) with literature…...……….…..…………..………..……4

Table 3S: The surface energy (γ_{100}, J m^{-2}) for models of the (100) surface with 2, 4, 6 and 8 layer slab thickness and varying constraints on the atoms furthest from the active surface..…...5

Table 4S: The entropy (S) and heat capacity (C_p) of molecules as derived in this work and compared to the literature [10,11]…………………………………………………………...6

Table 5S: The energy barrier, reaction energy, sticking coefficient (S_0), pre-exponential factor (A_0) and rate constant (k) at 300 K, 400 K and 500 K, for all considered reactions. The reaction numbers are of the format *X.Y* where *Y* represents all the reactions considered in the microkinetic model for reaction *X* of the Figure 1 in the main article……………….….…….7

Figure 1S: The surface energy (γ_{hkl}) of different surface facets, with Miller indices (*hkl*). Results from this study (green) are compared with previous computational studies (red) conducted using projector augmented wavefunction (PAW) method with the PBE functional [12]...……………………………………………………10

Figure 2S: The initial and optimized configuration of HCOOH when adsorbed on the β-Mo_2C surface in the *atop, bridge* and *C-hcp* sites. E_{ads} is given in each case in blue font. Atom colours are as in Figure 5 of the manuscript. ……………………….…………………………...11

Figure 3S: The initial and optimized configuration of HCOOH when adsorbed on the β-Mo_2C surface in the *fcc* and Mo-*hcp* sites. E_{ads} is given in each case in blue font. Atom colours are as in Figure 5 of the manuscript..………………………………………………………..12

Figure 4S: Adsorption configurations of CO_2 on the β-Mo_2C surface for each site and molecular configuration: (a) *atop*, *vertical*; (b) *bridge, lateral*; (c) *bridge*, *vertical*; (d) C-*hcp*, *lateral*; (e) *atop*, *lateral*; (f) *fcc*, *vertical*; (g) C-*hcp*, *vertical*; (h) Mo-*hcp*, *lateral*; (i) Mo-*hcp*, vertical; (j) *fcc, lateral*. Atom colours are as in Figure 5 of the manuscript………………………………..13

Figure 5S: Configurations of CO_2 on the β-Mo_2C surface for each site and molecular configuration: (a) *atop*, *vertical*; (b) *bridge, lateral*; (c) *bridge*, *vertical*; (d) C-*hcp*, *lateral*; (e) *atop*, *lateral*; (f) *fcc*, *vertical*; (g) C-*hcp*, *vertical*; (h) Mo-*hcp*, *lateral*; (i) Mo-*hcp*, vertical; (j) *fcc, lateral*. Atom colours are as in Figure 5 of the manuscript..…………………………......14

Figure 6S: Configurations of H_2O adsorbed on β-Mo_2C at different catalyst sites, in different orientations, as labelled. Atom colours are as in Figure 5 of the manuscript………………….15

Figure 7S: Optimized models of reactants, transition state and products on the β-Mo_2C (100) surface for reactions 1 through 7. Atom colours are as in Figure 5 of the manuscript...…...…...16

Figure 8S: Optimized models of reactants, transition state and products on the β-Mo_2C (100) surface for reactions 8 through 12. Atom colours are as in Figure 5 of the manuscript……….17

References……………………………………………………………………………18

Table 1S: Convergence testing of **k**-grid sampling. The energy change (ΔE) as a function of sampling is obtained for a unit cell of x = 4.754 Å, y=5.241 Å and z= 6.076 Å, with the selected converged settings highlighted in grey.

**k**-point density (Å^{-1})	**k**-grid (*x*-axis)	**k**-grid (y-axis)	**k**-grid (z-axis)	Energy (eV)	ΔE (eV)
0.01	21	19	16	-894028.7805	-
0.02	11	10	8	-894028.7783	0.0022
0.03	7	6	5	-894028.7875	-0.0092
0.04	5	5	4	-894028.7916	-0.0040
0.05	4	4	3	-894028.7583	0.0333
0.06	4	3	3	-894028.7466	0.0117
0.07	3	3	2	-894028.6356	0.1110
0.08	3	2	2	-894028.5719	0.0637
0.09	2	2	2	-894028.9971	-0.4251
0.10	2	2	2	-894028.9971	0.0000

Table 2S: Comparison of the formation energy (E_{form}), cohesive energy (E_{coh}), bulk volume and lattice parameters calculated in this work for different functionals (PBE [1,2], M06-L [3], PBE0 [4,5] and B3LYP [6]) (*light* basis) with literature.

	Formation energy (eV)	Cohesive energy (eV)	Bulk Volume ($Å^{-3}$)	X (Å)	Y (Å)	Z (Å)
Computational Literature [7]	-0.46	-	-	-	-	-
Computational literature [8]	-0.30	-21.00	-	-	-	-
Experimental Literature [9]	-	-	147.46	4.724	5.199	6.004
PBE+TS	-0.74	-23.94	143.84	4.685	5.149	5.963
PBE	-1.74	-21.17	150.43	4.740	5.233	6.065
M06-L	-1.90	-22.40	149.48	4.769	5.192	6.037
B3LYP	Geometry optimisation unconverged after 8x computational cost					
PBE0	Geometry optimisation unconverged after 8x computational cost					

Definition of derived energies:

The cohesive energy (E_{coh}) of the optimized β-Mo_2C bulk unit cell is calculated as:

$$E_{\mathrm{coh}} = \frac{E_{\mathrm{Mo_2C}}^{\mathrm{bulk}} - n \cdot E_{\mathrm{Mo}}^{\mathrm{atom}} - m \cdot E_{\mathrm{C}}^{\mathrm{atom}}}{b}$$

where $E_{\mathrm{Mo_2C}}^{\mathrm{bulk}}$ is the energy of the bulk unit cell, $E_{\mathrm{Mo}}^{\mathrm{atom}}$ and $E_{\mathrm{C}}^{\mathrm{atom}}$ are the energy of a gas-phase molybdenum and carbon atom, respectively, n and m are the number of molybdenum and carbon atoms, respectively, and b is the number of units of Mo_2C in the bulk model [defined as $b = (n+m)/3$]. Similarly, the formation energy (E_{form}) of the β-Mo_2C structure is calculated as:

$$E_{\mathrm{form}} = E_{\mathrm{Mo_2C}}^{\mathrm{bulk}} - n \cdot E_{\mathrm{Mo}}^{\mathrm{bulk}} - m \cdot E_{\mathrm{C}}^{\mathrm{bulk}}$$

where $E_{\mathrm{Mo}}^{\mathrm{bulk}}$ and $E_{\mathrm{C}}^{\mathrm{bulk}}$ are the energy of a bulk Mo (bcc) and carbon atom (graphite), respectively, both in their respective ground state structures.

Table 3S: The surface energy (γ_{100}, J m^{-2}) for (1 x 1) models of the (100) surface with 2, 4, 6 and 8 layer slab thickness and varying constraints on the atoms furthest from the active surface.

Unconstrained Layers \ Slab thickness (layers)	2	4	6	8
0	4.88	5.24	5.34	5.38
1	3.95	5.00	5.13	5.18
2	3.10	4.99	5.10	5.15
3	-	4.59	5.09	5.14
4	-	4.53	5.08	5.13
5	-	-	4.74	5.12
6	-	-	4.71	5.12
7	-	-	-	4.80
8	-	-	-	4.79

Table 4S: The entropy (S) and heat capacity (C_p) of molecules as derived in this work and compared to the literature [10,11].

Gas phase species	T (K)	C_p (J mol^{-1} K^{-1})		S (J mol^{-1} K^{-1})		$H - H_{298.15}$ (kJ mol^{-1})	
		This work	Literature	This work	Literature	This work	Literature
CO	298.00	-	29.15	-	-	-	-
	300.00	28.95	29.15	196.91	197.84	0.00	0.06
	400.00	28.95	29.30	205.24	206.24	1.93	2.97
	500.00	28.95	29.82	211.70	212.83	4.05	5.93
H_2	298.00	-	28.84	-	130.67	-	-
	300.00	28.95	28.85	135.68	130.86	0.00	0.05
	400.00	28.95	29.18	144.01	139.22	1.33	2.96
	500.00	28.95	29.26	150.47	145.74	2.87	5.88
H_2O	298.00	-	33.59	-	188.82	-	-
	300.00	33.31	33.60	193.93	189.04	0.00	0.06
	400.00	33.96	34.26	203.59	198.79	1.57	3.45
	500.00	34.91	35.22	211.27	206.53	3.77	6.92
CO_2	298.00	-	37.12	-	213.79	-	-
	300.00	33.27	37.22	216.24	214.02	0.74	0.07
	400.00	35.30	41.34	226.10	225.31	2.37	4.00
	500.00	36.96	44.61	234.16	234.90	4.69	8.31
HCOOH	298.00	-	45.68 ± 0.07	-	248.70	-	-
	300.00	44.54	45.84	252.30	-	-	-
	400.00	51.66	54.52	266.11	-	-	-
	500.00	57.33	62.63	278.28	-	-	-

Table 5S: The energy barrier, reaction energy, sticking coefficient (S_0), pre-exponential factor (A_0) and rate constant (k) at 300 K, 400 K and 500 K, for all considered reactions. The reaction numbers are of the format *X.Y* where *Y* represents all the reactions considered in the microkinetic model for reaction *X* of the Figure 1 in the main article.

Reaction number	Reaction	Energy barrier (eV)	Reaction energy (eV)	300 K			400 K			500 K		
				S_0	A_0	k (s^{-1})	S_0	A_0	k (s^{-1})	S_0	A_0	k (s^{-1})
1.1	HCOOH + * → HCOOH*	-	-2.20	7.94E-08	6.87E+03	5.46E-04	3.87E-08	5.95E+03	2.30E-04	2.21E-08	5.32E+03	1.18E-04
1.2	HCOOH* → HCOOH + *	-	2.20	-	8.99E+11	1.88E-05	-	3.20E+11	1.29E-03	-	1.26E+11	9.65E-02
2.1	HCOOH* → HCOO-H*	0.51	-0.80	-	8.54E+12	8.44E+08	-	7.78E+12	5.50E+08	-	7.20E+12	3.70E+08
2.2	HCOO-H* → HCOOH*	1.32	0.80	-	1.38E+13	1.41E+00	-	1.45E+13	1.38E+00	-	1.49E+13	1.37E+00
2.3	HCOO-H* + * → HCOO* + H*	-	-0.27	-	1.28E+13	1.03E+16	-	1.33E+13	1.07E+16	-	1.38E+13	1.13E+16
2.4	HCOO* + H* → HCOO-H* + *	-	0.27	-	8.46E+12	1.05E+10	-	8.15E+12	1.02E+10	-	7.88E+12	9.64E+09
3.1	HCOOH* → H-COOH*	0.27	-0.72	-	1.08E+13	1.19E+11	-	1.13E+13	1.01E+11	-	1.17E+13	9.00E+10
3.2	H-COOH* → HCOOH*	0.99	0.72	-	1.51E+13	2.76E+03	-	1.68E+13	3.05E+03	-	1.78E+13	3.35E+03
3.3	H-COOH* + * → H* + COOH*	-	-0.10	-	1.06E+13	1.49E+14	-	1.08E+13	1.48E+14	-	1.10E+13	1.51E+14
3.4	H* + COOH* → H-COOH*	-	0.10	-	1.03E+13	7.28E+11	-	1.01E+13	7.32E+11	-	9.85E+12	7.20E+11
4.1	HCOOH* → HCO-OH*	0.34	-1.23	-	1.36E+13	9.58E+09	-	1.45E+13	9.36E+09	-	1.50E+13	9.38E+09
4.2	HCO-OH* → HCOOH*	1.58	1.23	-	1.97E+13	3.13E-03	-	1.91E+13	3.11E-03	-	1.78E+13	2.88E-03
4.3	HCO-OH* + * → HCO* + OH*	-	-0.13	-	1.17E+13	4.10E+14	-	1.20E+13	4.18E+14	-	1.21E+13	4.21E+14
4.4	HCO* + OH* → HCO-OH* + *	-	0.13	-	9.25E+12	2.65E+11	-	9.06E+12	2.60E+11	-	8.95E+12	2.58E+11
5.1	HCOO* → H-COO*	0.38	-0.43	-	9.78E+12	7.55E+09	-	1.00E+13	6.17E+09	-	1.02E+13	5.28E+09
5.2	H-COO* → HCOO*	0.81	0.43	-	1.42E+13	1.50E+05	-	1.50E+13	1.55E+05	-	1.53E+13	1.59E+05
5.3	H-COO* + * → H* + CO_2*	-	-0.13	-	1.42E+13	4.06E+14	-	1.50E+13	4.43E+14	-	1.57E+13	4.86E+14
5.4	H* + CO_2* → H-COO*	-	0.13	-	7.65E+12	2.67E+11	-	7.23E+12	2.45E+11	-	6.92E+12	2.23E+11
6.1	HCOO*+ H* → HCOO*-H* + *	-	0.27	-	8.46E+12	1.05E+10	-	8.15E+12	1.02E+10	-	7.87E+12	9.62E+09
6.2	HCOO*-H* + * → HCOO* + H*	-	-0.27	-	1.28E+13	1.03E+16	-	1.33E+13	1.07E+16	-	1.38E+13	1.13E+16
6.3	HCOO*-H* → H2-COO*	1.35	1.25	-	8.66E+13	8.59E+00	-	1.61E+14	2.11E+01	-	2.58E+14	5.48E+01
6.4	H2-COO* → HCOO*-H*	0.09	-1.25	-	1.12E+13	1.34E+12	-	1.08E+13	1.28E+12	-	1.04E+13	1.23E+12
6.5	H2-COO* → H_2 + CO_2*	-	0.12	-	1.47E+12	7.63E+13	-	7.07E+11	5.00E+14	-	3.76E+11	3.87E+15

6.6	H_2 + CO_2* → H2-COO*	-	-0.12	-	3.69E+08	7.10E+06	-	2.80E+08	3.96E+05	-	2.41E+08	2.34E+04
7.1	COOH* → COO-H*	0.74	-0.68	-	8.59E+12	4.77E+06	-	8.00E+12	3.16E+06	-	7.54E+12	2.17E+06
7.2	COO-H* → COOH*	1.43	0.68	-	1.19E+13	9.85E-02	-	1.22E+13	9.19E-02	-	1.23E+13	8.67E-02
7.3	COO-H* + * → CO_2* + H*	-	-0.13	-	1.42E+13	4.05E+14	-	1.50E+13	4.42E+14	-	1.57E+13	4.85E+14
7.4	CO_2* + H* → COO-H*	-	0.13	-	7.65E+12	2.68E+11	-	7.23E+12	2.46E+11	-	6.92E+12	2.24E+11
8.1	COOH* → CO-OH*	0.24	-1.52	-	1.23E+13	7.24E+10	-	1.25E+13	6.79E+10	-	1.24E+13	6.41E+10
8.2	CO-OH* → COOH*	1.76	1.52	-	1.26E+13	2.18E-05	-	1.14E+13	1.87E-05	-	1.03E+13	1.54E-05
8.3	CO-OH* + * → CO* + OH*	-	-0.07	-	1.96E+13	1.24E+14	-	2.20E+13	1.59E+14	-	2.39E+13	1.99E+14
8.4	CO* + OH* → CO-OH*	-	0.07	-	5.55E+12	8.78E+11	-	4.93E+12	6.83E+11	-	4.54E+12	5.44E+11
9.1	H* + COOH* → H*-COOH* + *	-	0.10	-	1.03E+13	7.28E+11	-	1.01E+13	7.32E+11	-	9.85E+12	7.20E+11
9.2	H*-COOH* + * → H* + COOH*	-	-0.10	-	1.06E+13	1.49E+14	-	1.08E+13	1.48E+14	-	1.10E+13	1.51E+14
9.3	H*-COOH* → CO*-H_2O*	1.03	0.23	-	3.57E+13	1.02E+04	-	4.92E+13	1.52E+04	-	6.23E+13	2.32E+04
9.4	CO*-H_2O* → H*-COOH*	0.80	-0.23	-	3.68E+12	6.44E+05	-	3.02E+12	2.98E+05	-	2.64E+12	1.54E+05
9.5	CO*-H_2O* → CO* + H_2O*	-	-0.07	-	1.96E+13	1.24E+14	-	2.20E+13	1.59E+14	-	2.39E+13	1.99E+14
9.6	CO* + H_2O* → CO*-H_2O*	-	0.07	-	5.55E+12	8.78E+11	-	4.93E+12	6.83E+11	-	4.54E+12	5.44E+11
10.1	HCO* → H-CO*	0.28	-0.81	-	1.86E+13	1.54E+11	-	2.13E+13	1.66E+11	-	2.32E+13	1.78E+11
10.2	H-CO* → HCO*	1.08	0.81	-	6.70E+12	1.08E+02	-	6.73E+12	9.26E+01	-	6.70E+12	8.03E+01
10.3	H-CO* + * → H* + CO*	-	-0.24	-	6.40E+12	1.48E+15	-	6.07E+12	1.26E+15	-	5.89E+12	1.11E+15
10.4	H* + CO* → H-CO*	-	0.24	-	1.69E+13	7.35E+10	-	1.79E+13	8.61E+10	-	1.84E+13	9.82E+10
11.1	OH*-H* → H_2O*	2.44	1.94	-	3.13E+13	2.53E-11	-	3.61E+13	3.19E-11	-	3.88E+13	3.83E-11
11.2	H_2O* → OH*-H*	0.50	-1.94	-	2.40E+12	2.62E+08	-	1.82E+12	1.06E+08	-	1.49E+12	4.69E+07
11.3	H_2O* + * → OH* + H*	-	-0.04	-	1.12E+13	3.31E+13	-	1.14E+13	3.42E+13	-	1.16E+13	3.53E+13
11.4	OH* + H* → H_2O* + *	-	0.04	-	9.72E+12	3.28E+12	-	9.52E+12	3.17E+12	-	9.37E+12	3.07E+12
12.1	H*-H* → H_2*	1.42	1.42	-	2.41E+13	2.00E-01	-	3.17E+13	3.06E-01	-	3.82E+13	4.55E-01
12.2	H_2* → H*-H*	0.01	-1.42	-	1.03E+13	1.97E+13	-	9.68E+12	1.57E+13	-	9.08E+12	1.26E+13
12.3	H_2* → H* + H*	-	-0.02	-	1.04E+13	1.67E+13	-	1.05E+13	1.67E+13	-	1.05E+13	1.68E+13
12.4	H* + H* → H_2*	-	0.02	-	1.04E+13	6.51E+12	-	1.04E+13	6.50E+12	-	1.03E+13	6.48E+12
13.1	CO_2* → CO_2 + *	-	1.76	-	1.26E+12	1.67E-01	-	5.03E+11	5.89E+00	-	2.17E+11	2.22E+02
13.2	CO_2 + * → CO_2*	-	-1.76	2.76E-06	5.44E+03	1.50E-02	1.55E-06	5.44E+03	8.45E-03	9.93E-07	5.44E+03	5.41E-03

14.1	$H_2O^* \rightarrow H_2O + *$	-	0.86	-	3.31E+11	1.18E+07	-	1.19E+11	1.60E+08	-	4.87E+10	2.58E+09
14.2	$H_2O + * \rightarrow H_2O^*$	-	-0.86	4.06E-05	8.51E+03	3.45E-01	1.98E-05	8.51E+03	1.68E-01	1.13E-05	8.51E+03	9.63E-02
15.1	$CO^* \rightarrow CO + *$	-	2.73	-	1.56E+12	2.33E-11	-	7.33E+11	6.52E-10	-	3.70E+11	2.04E-08
15.2	$CO + * \rightarrow CO^*$	-	-2.73	2.13E-05	6.82E+03	1.46E-01	1.20E-05	6.82E+03	8.19E-02	7.68E-06	6.82E+03	5.24E-02
16.1	$H_2^* \rightarrow H_2 + *$	-	0.77	-	4.19E+12	3.98E+07	-	2.63E+12	3.91E+08	-	1.69E+12	4.58E+09
16.2	$H_2 + * \rightarrow H_2^*$	-	-0.77	9.37E-03	2.54E+04	2.38E+02	5.27E-03	2.54E+04	1.34E+02	3.37E-03	2.54E+04	8.58E+01

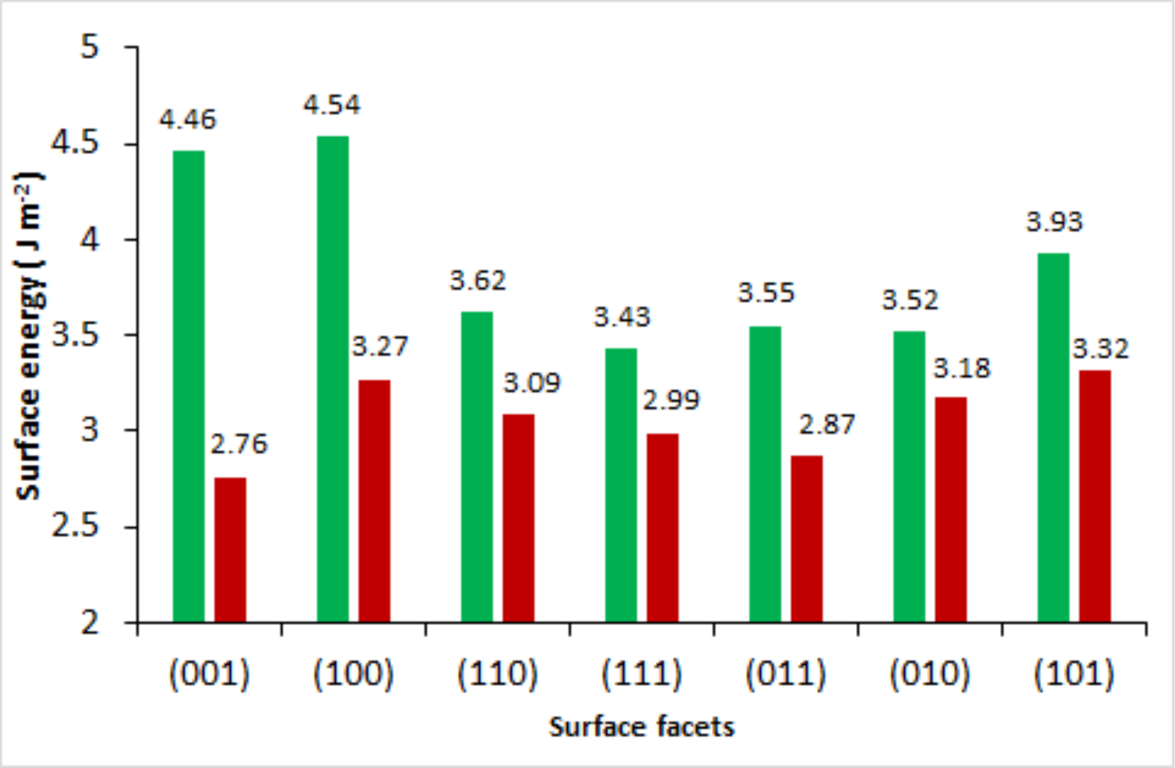

Figure 1S: The surface energy (γ_{hkl}) of different (2 x 2) surface facets, with Miller indices (*hkl*). Results from this study (green) are compared with previous computational studies (red) conducted using projector augmented wavefunction (PAW) method with the PBE functional [12].

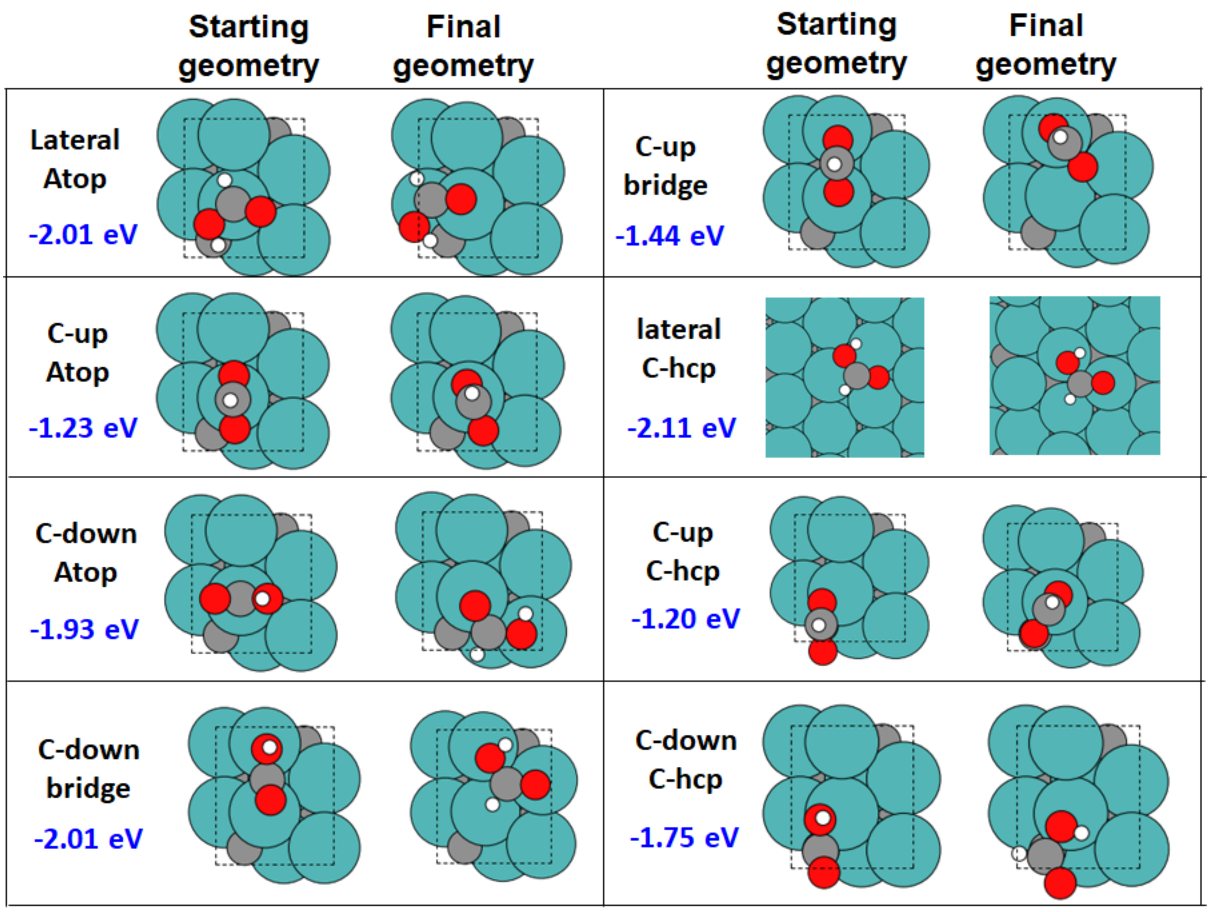

Figure 2S: The initial and optimized configuration of HCOOH when adsorbed on the β-Mo_2C surface in the *atop, bridge* and *C-hcp* sites. E_{ads} is given in each case in blue font. Atom colours are as in Figure 5 of the manuscript.

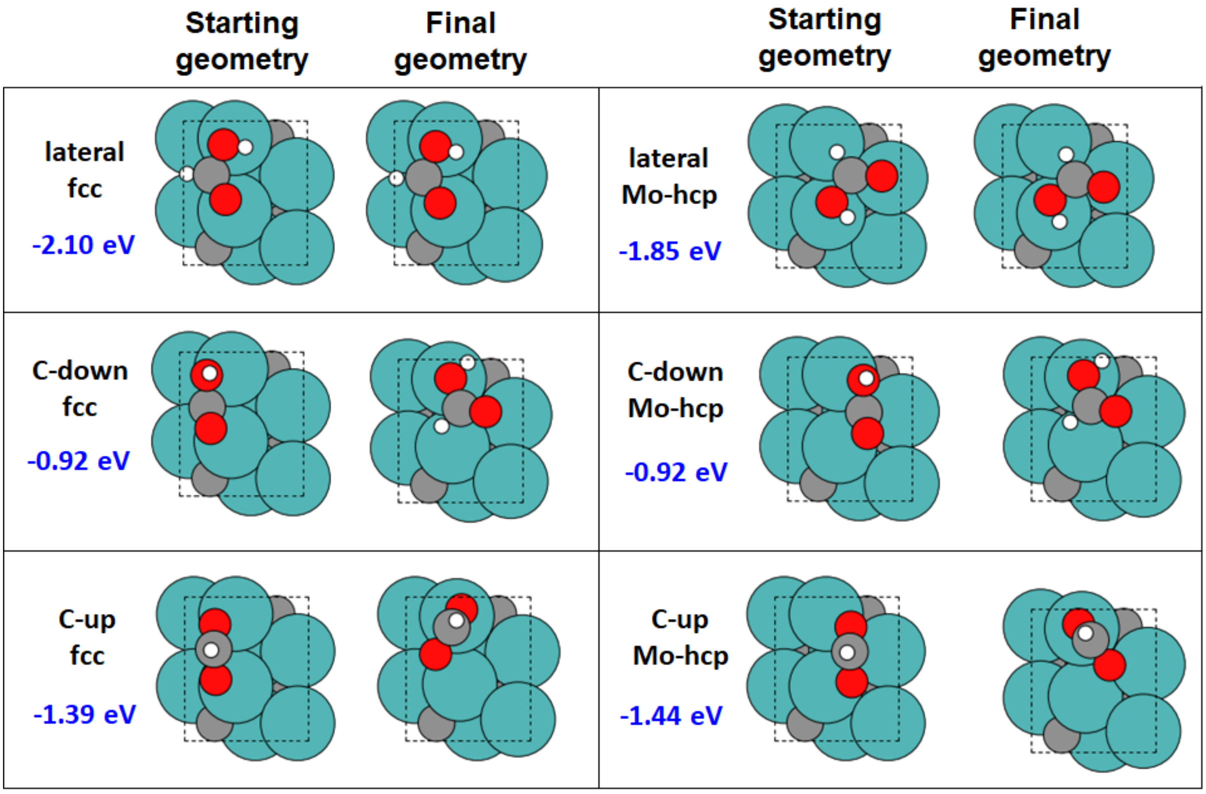

Figure 3S: The initial and optimized configuration of HCOOH when adsorbed on the β-Mo_2C surface in the *fcc* and Mo-*hcp* sites. E_{ads} is given in each case in blue font. Atom colours are as in Figure 5 of the manuscript.

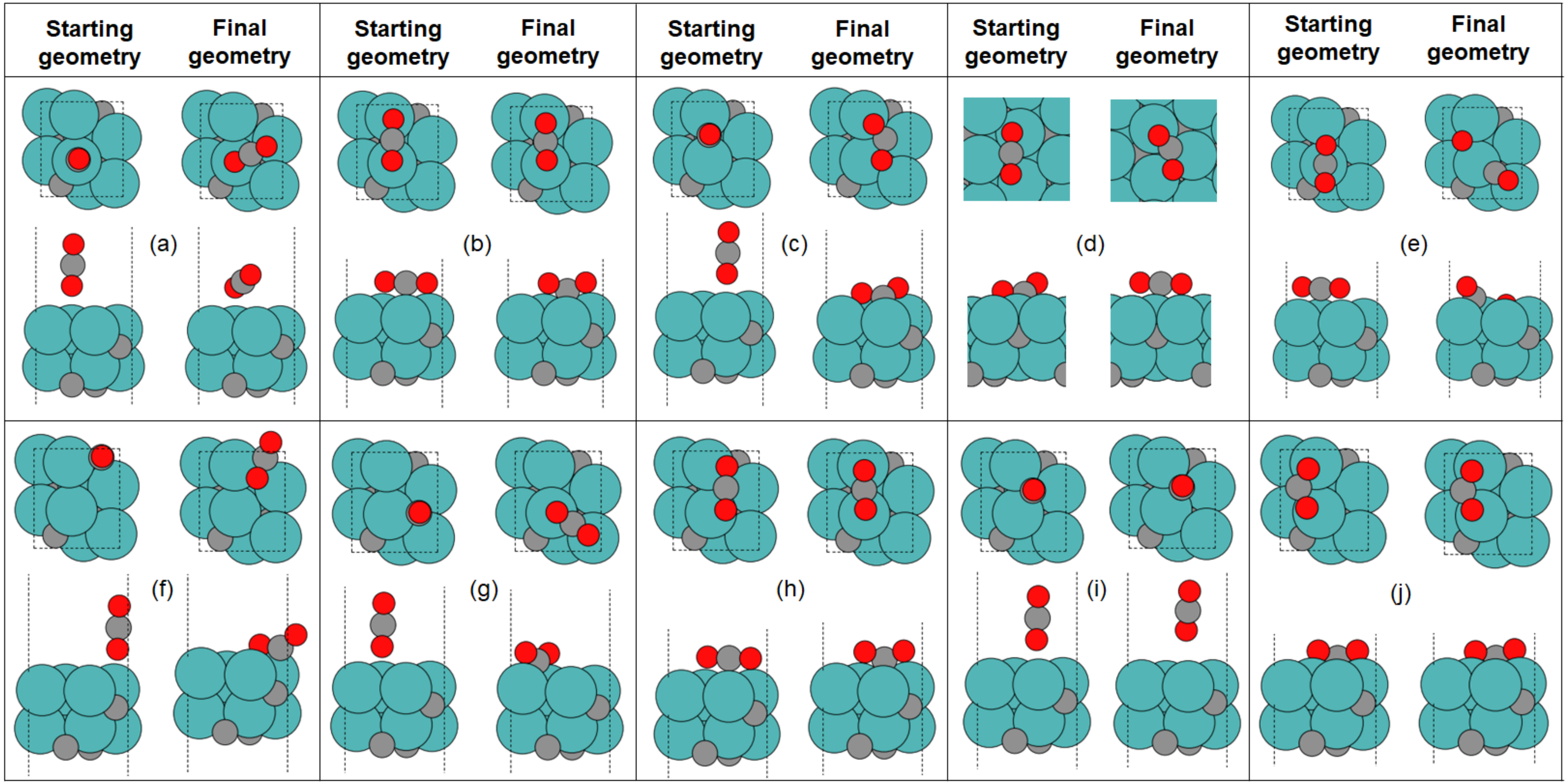

Figure 4S: Configurations of CO_2 on the β-Mo_2C surface for each site and molecular configuration: (a) *atop*, *vertical*; (b) *bridge, lateral*; (c) *bridge*, *vertical*; (d) C-*hcp*, *lateral*; (e) *atop*, *lateral*; (f) *fcc*, *vertical*; (g) C-*hcp*, *vertical*; (h) Mo-*hcp*, *lateral*; (i) Mo-*hcp*, vertical; (j) *fcc, lateral*. Atom colours are as in Figure 5 of the manuscript.

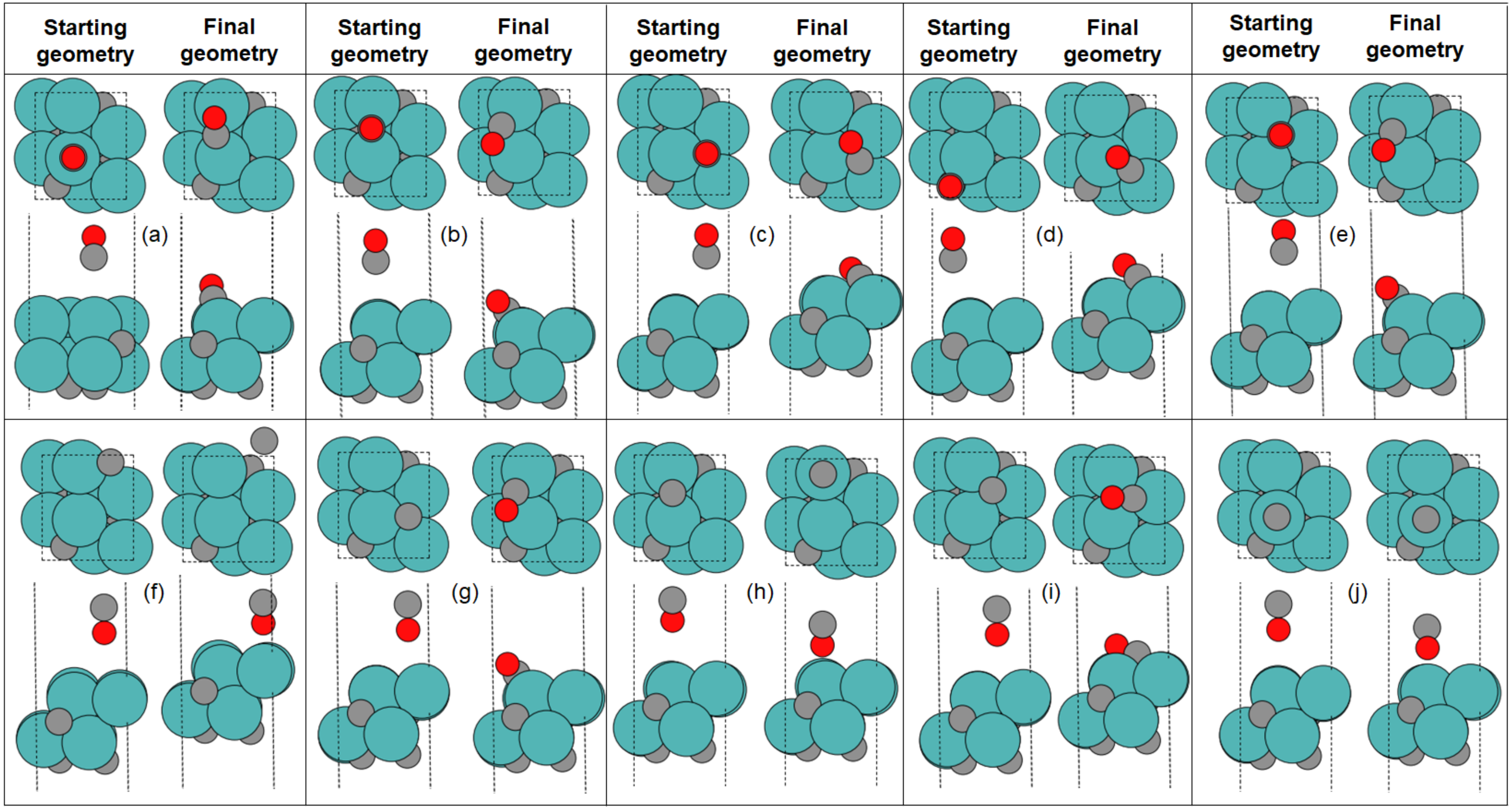

Figure 5S: Configurations of CO on the β-Mo_2C surface for each site and molecular configuration (a) *atop*, *C-down*; (b) *bridge*, *C-down*; (c) C-*hcp*, *C-down*; (d) Mo-*hcp*, *C-down*; (e) *fcc*, *C-down*; (f) *fcc*, *C-up*; (g) C-*hcp*, *C-up*; (h) *bridge*, *C-up*; (i) Mo-*hcp*, *C-up*; (j) *atop*, *C-down*. Atom colours are as in Figure 5 of the manuscript.

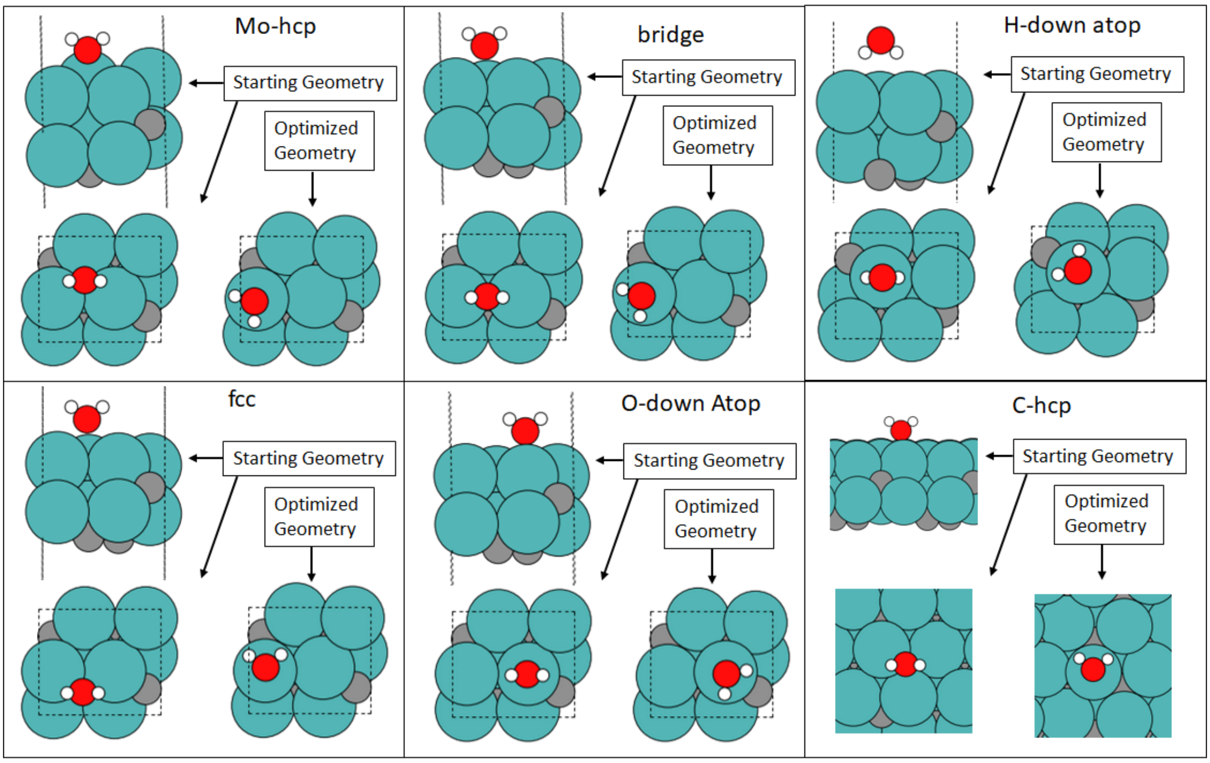

Figure 6S: Configurations of H_2O adsorbed on β-Mo_2C at different catalyst sites, in different orientations, as labelled. Atom colours are as in Figure 5 of the manuscript.

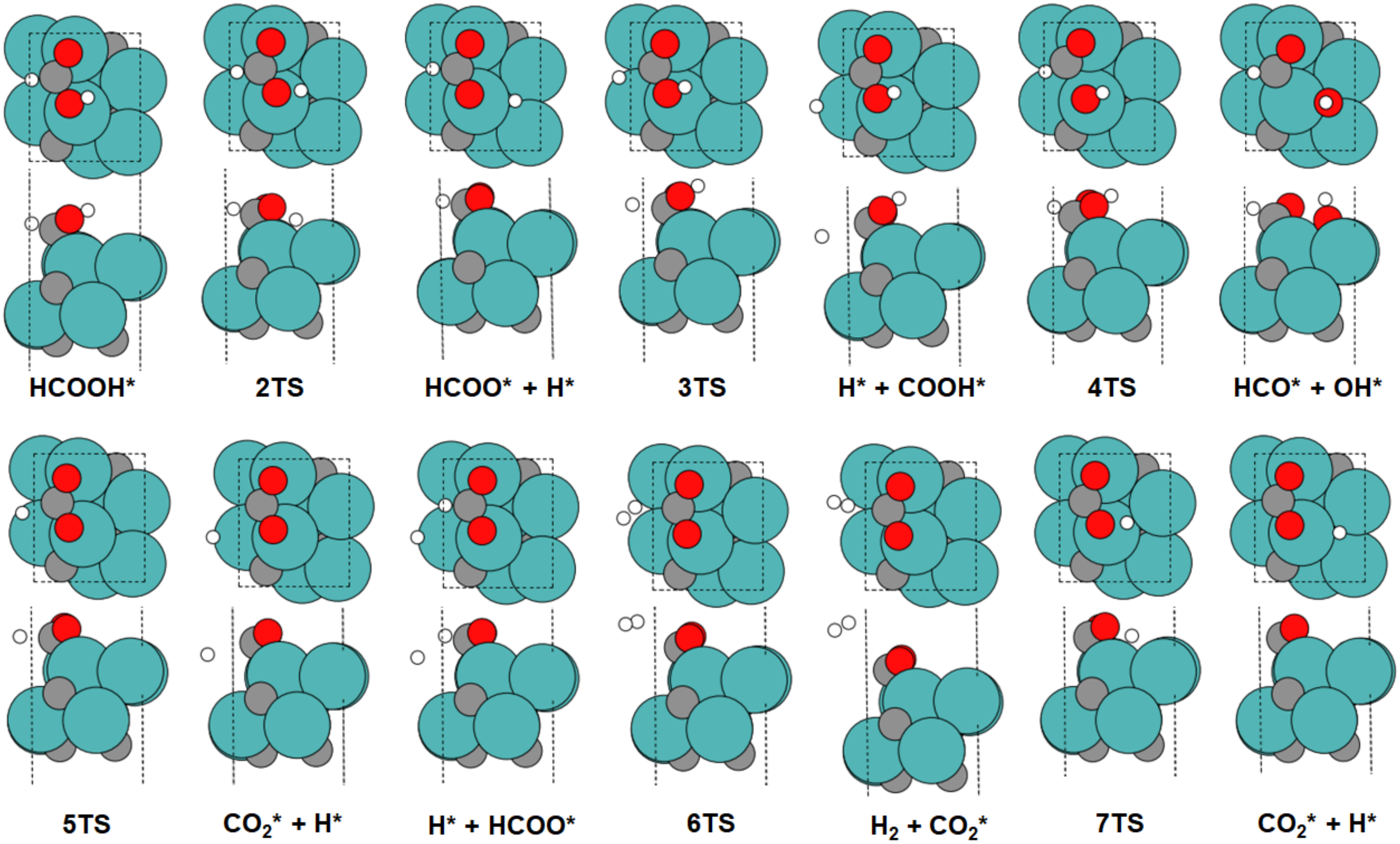

Figure 7S: Optimized models of reactants, transition state and products on the β-Mo_2C (100) surface for reactions 1 through 7. Atom colours are as in Figure 5 of the manuscript.

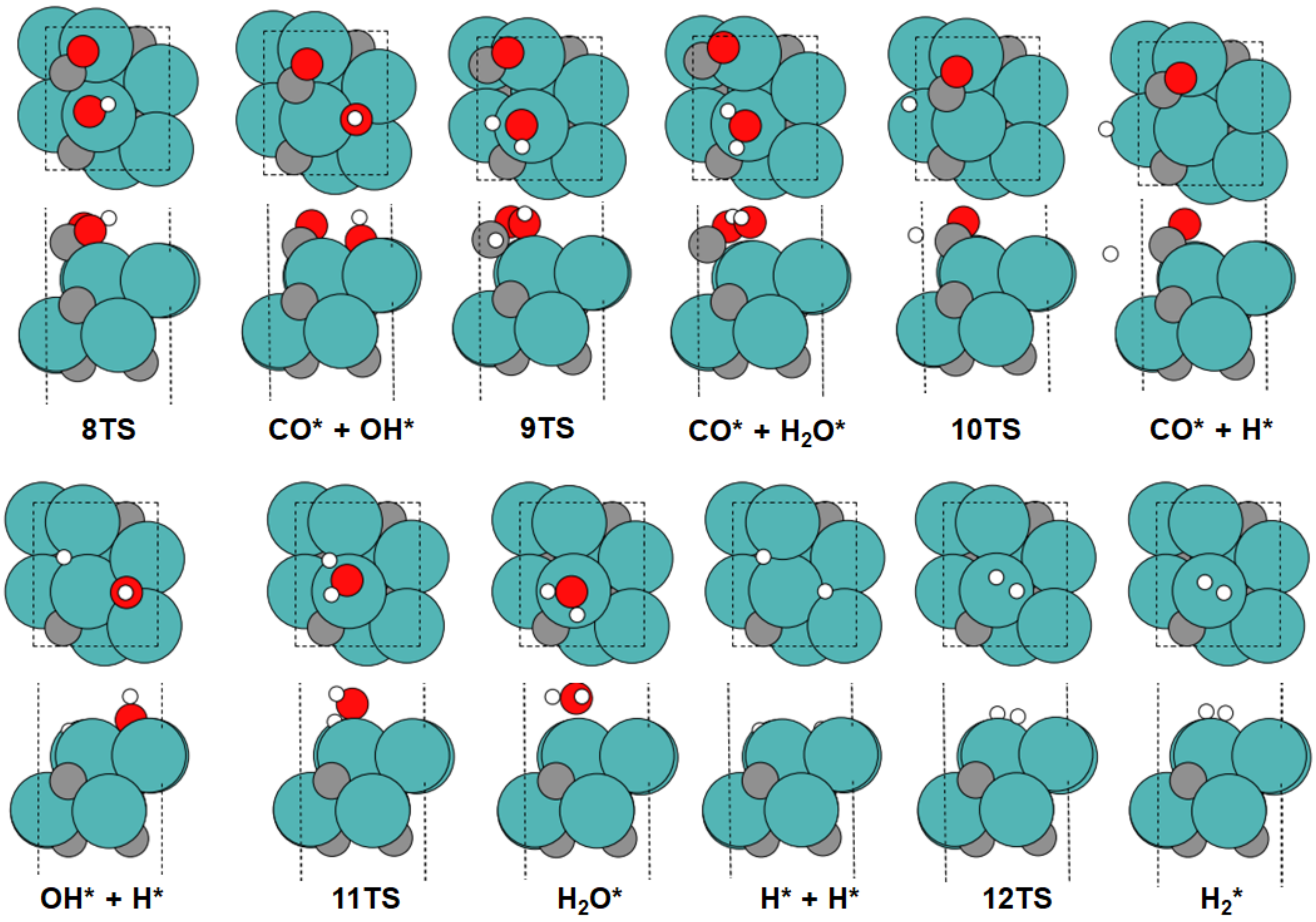

Figure 8S: Optimized models of reactants, transition state and products on the β-Mo_2C (100) surface for reactions 8 through 12. Atom colours are as in Figure 5 of the manuscript.

References:

[1] J.P. Perdew, K. Burke, M. Ernzerhof, Generalized Gradient Approximation Made Simple, Phys. Rev. Lett. 77 (1996) 3865–3868. https://doi.org/10.1103/PhysRevLett.77.3865.

[2] J.P. Perdew, K. Burke, M. Ernzerhof, Generalized Gradient Approximation Made Simple [Phys. Rev. Lett. 77, 3865 (1996)], Phys. Rev. Lett. 78 (1997) 1396–1396. https://doi.org/10.1103/PhysRevLett.78.1396.

[3] Y. Zhao, D.G. Truhlar, A new local density functional for main-group thermochemistry, transition metal bonding, thermochemical kinetics, and noncovalent interactions, J. Chem. Phys. 125 (2006) 194101. https://doi.org/10.1063/1.2370993.

[4] J.P. Perdew, M. Ernzerhof, K. Burke, Rationale for mixing exact exchange with density functional approximations, J. Chem. Phys. 105 (1996) 9982–9985. https://doi.org/10.1063/1.472933.

[5] C. Adamo, V. Barone, Toward reliable density functional methods without adjustable parameters: The PBE0 model, J. Chem. Phys. 110 (1999) 6158–6170. https://doi.org/10.1063/1.478522.

[6] A.D. Becke, A new mixing of Hartree–Fock and local density-functional theories, J. Chem. Phys. 98 (1993) 1372–1377. https://doi.org/10.1063/1.464304.

[7] F.Z. Abderrahim, H.I. Faraoun, T. Ouahrani, Structure, bonding and stability of semi-carbides M2C and sub-carbides M4C (M=V, Cr, Nb, Mo, Ta, W): A first principles investigation, Phys. B Condens. Matter. 407 (2012) 3833–3838. https://doi.org/10.1016/j.physb.2012.05.070.

[8] J.R.D.S. Politi, F. Viñes, J.A. Rodriguez, F. Illas, Atomic and electronic structure of molybdenum carbide phases: Bulk and low Miller-index surfaces, Phys. Chem. Chem. Phys. 15 (2013) 12617–12625. https://doi.org/10.1039/c3cp51389k.

[9] E. Parthé, V. Sadagopan, The structure of dimolybdenum carbide by neutron diffraction technique, Acta Crystallogr. 16 (1963) 202–205. https://doi.org/10.1107/S0365110X63000487.

[10] M. Chase, NIST-JANAF Thermochemical Tables, 4th Edition, J. Phys. Chem. Ref.

Data, Monogr. 9. (1998).

[11] J. Chao, K.R. Hall, K.N. Marsh, R.C. Wilhoit, Thermodynamic Properties of Key Organic Oxygen Compounds in the Carbon Range C1 to C4. Part 2. Ideal Gas Properties, J. Phys. Chem. Ref. Data. (1986). https://doi.org/10.1063/1.555769.

[12] T. Wang, Q. Luo, Y.W. Li, J. Wang, M. Beller, H. Jiao, Stable surface terminations of orthorhombic Mo2C catalysts and their CO activation mechanisms, Appl. Catal. A Gen. 478 (2014) 146–156. https://doi.org/10.1016/j.apcata.2014.03.042.
